# The suppressive efficacy of THZ1 depends on *KRAS* mutation subtype and is associated with super‐enhancer activity and the PI3K/AKT/mTOR signalling in pancreatic ductal adenocarcinoma: A hypothesis‐generating study

**DOI:** 10.1002/ctm2.1500

**Published:** 2023-11-30

**Authors:** Lei Huang, Hui Yang, Kaidi Chen, Jing Yuan, Jie Li, Guanghai Dai, Mancang Gu, Yan Shi

**Affiliations:** ^1^ Department of Oncology Ruijin Hospital Shanghai Jiao Tong University School of Medicine Shanghai China; ^2^ School of Pharmaceutical Science Zhejiang Chinese Medical University Hangzhou China; ^3^ Department of Pathology Chinese PLA General Hospital Beijing China; ^4^ Department of Medical Oncology Chinese PLA General Hospital Beijing China; ^5^ Academy of Chinese Medical Sciences Zhejiang Chinese Medical University Hangzhou China; ^6^ Department of General Surgery Shanghai Seventh People's Hospital Shanghai University of Traditional Chinese Medicine Shanghai China

**Keywords:** cyclin‐dependent kinase 7, *KRAS* mutants, pancreatic ductal adenocarcinoma, PI3K/AKT/mTOR signalling, precision oncology, RNA polymerase II, super‐enhancer, THZ1

## Abstract

**Background:**

Inhibition of CDK7, a potent transcription regulator, may bring new hope for treating pancreatic ductal adenocarcinoma (PDAC), which is featured by large genetic heterogeneity and abundant *KRAS* mutations. This investigation aimed at exploring the discrepant efficacies of THZ1, a small‐molecule covalent CDK7 inhibitor, on PDACs with different *KRAS* mutations and the underlying mechanisms.

**Methods:**

Associations of CDK7 expression with survival by *KRAS* mutations were first assessed. Effects of THZ1 on PDAC by different *KRAS* mutations were then investigated in vitro and in vivo. Moreover, the effects of THZ1 on gene transcription and phosphorylation of RNA polymerase II (RNAPOLII) in different *KRAS* mutant PDACs were assessed, and the effect of THZ1 on super‐enhancer activity was evaluated using chromatin immunoprecipitation sequencing. Lastly, the effects of THZ1 on the binding of H3K27ac to *PIK3CA* and on the PI3K/AKT/mTOR signalling were analysed.

**Results:**

High CDK7 expression was significantly linked to worse survival within PDAC patients carrying *KRAS‐G12V* mutation but not in those with *KRAS‐G12D* mutation. The apoptosis‐inducing effect of THZ1 was markedly stronger in *KRAS‐G12V* PDAC than *KRAS‐G12D* cancer. THZ1 significantly inhibited the growth of xenograft tumour with *KRAS‐G12V* mutation, and the inhibition was markedly stronger than for *KRAS‐G12D* tumour. In mini‐cell‐derived xenograft (CDX) models, THZ1 significantly suppressed *KRAS‐G12V* PDAC but not *KRAS‐G12D* cancer. THZ1 significantly suppressed the phosphorylation of RNAPOLII, and this effect was stronger in *KRAS‐G12V* PDAC (especially at ser5). *KRAS‐G12V* PDAC had more H3K27ac‐binding super‐enhancers, and the inhibition of THZ1 on super‐enhancer activity was also stronger in *KRAS‐G12V* PDAC. Furthermore, THZ1 significantly weakened the binding of H3K27ac to *PIK3CA* in *KRAS‐G12V* PDAC. THZ1 significantly suppressed the PI3K/AKT/mTOR pathway and its downstream markers, and this effect was stronger in *KRAS‐G12V* cells.

**Conclusions:**

In this hypothesis‐generating study, THZ1 might selectively inhibit certain PDACs with *KRAS‐G12V* mutation more potently compared with some other PDACs with *KRAS‐G12D* mutation, which might be associated with its effect on super‐enhancer activity and the PI3K/AKT/mTOR signalling. Our findings might offer novel key clues for the precise management of PDAC and important evidence for future targeted trial design.

**Highlights:**

- THZ1 had a stronger effect on PDAC‐bearing KRAS‐G12V mutation than G12D mutation.
- Suppressive effect of THZ1 on phosphorylation of RNAPOLII was stronger in KRAS‐G12V than KRAS‐G12D PDAC.
- Inhibition of THZ1 on super‐enhancer activity and H3K27ac binding to PIK3CA was stronger in KRAS‐G12V PDAC.
- Suppressive effect of THZ1 on PI3K/AKT/mTOR pathway was stronger in KRAS‐G12V PDAC.

## Graphical abstract

In this hypothesis‐generating study, the small‐molecule covalent CDK7 inhibitor THZ1 might selectively inhibit certain pancreatic ductal adenocarcinomas (PDACs) with KRAS‐G12V mutation more potently compared with some other PDACs with KRAS‐G12D mutation, which might be associated with its effect on super‐enhancer activity and the PI3K/AKT/mTOR‐signalling. Our findings might offer novel key clues for precise management of PDAC and important evidence for future targeted trial design.

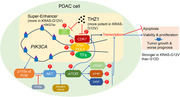

## 1 INTRODUCTION

Approximately 496 000 newly diagnosed pancreatic cancer (PaC) cases were reported globally in 2020, resulting in approximately 466 000 deaths. PaC ranks seventh as the leading cause of cancer‐associated mortality within both sexes, and PaC incidence has been increasing.^1^, ^2^, ^3^ PaCs, the majority of which are pancreatic ductal adenocarcinomas (PDACs), have a dismal prognosis. The 3‐year overall survival (OS) rate ranges from 9% to 34% for TNM Stage I–II cancers, and from less than 1% to 5% for Stage III–IV cancers.^3^ Although chemotherapy is the primary and major nonsurgical treatment for PDACs with definitive efficacy in prolonging survival, a significant proportion of such patients may experience rapid progression even with chemotherapy and/or molecular‐targeted therapies. This underscores the urgent need for novel effective and precise treatment options based on novel targets.^4^, ^5^

Targeted transcriptome regulation presents a promising avenue for PDAC treatment. Aberrant, sustained gene transcription activated by super‐enhancers (SEs) significantly correlates with cancer growth, progression and metastasis.^6^, ^7^, ^8^ This makes SE‐mediated transcription dysregulation an attractive target for anticancer therapy, particularly in cancers lacking reliable targets.^9^, ^10^, ^11^ Some cyclin‐dependent kinases (CDKs; e.g. CDK7, CDK8, CDK9, CDK12 and CDK13) regulate transcription by modulating the phosphorylation of the carboxy‐terminal domain (CTD) of RNA polymerase II (RNAPOLII).^12^, ^13^, ^14^, ^15^, ^16^, ^17^, ^18^ The CDK family can be harnessed to regulate oncogene activity.

CDK7 has been revealed to have a dual role in the regulation of transcription and cell cycle.^19^ PaC is highly addictive to CDK7‐dependent transcription.^20^ Inhibiting CDK7 potently and selectively weakens the proliferation and viability of human PDAC cells, and represses PDAC progression in preclinical models, by pronouncedly downregulating transcription of genes, including those linked to the NF‐κB signalling and mitotic cell cycle.^20^ CDK7 inhibition causes DNA damage, cell cycle arrest and apoptosis, and strengthens multidrug chemotherapy response within PDAC.^21^ CDK7 emerges as a potential anti‐PDAC target, but designing highly specific CDK7 inhibitors (CDK7is) is a major challenge. Targeting cysteine residues outside the ATP‐binding cassette of CDK7 is a novel approach for developing CDK7is,^22^ enabling selective repression of CDK7 kinase activity, RNAPOLII CTD phosphorylation, SE activity and gene transcription, culminating in cancer cell death.^22^, ^23^, ^24^ THZ1, a covalent small‐molecule CDK7 inhibitor that also exerts inhibition on CDK12 and CDK13,^25^, ^26^, ^27^ was developed by the Gray Lab in 2014.^22^ It has demonstrated promising anticancer potential across a spectrum of treatment‐resistant malignancies, encompassing *MYCN*‐amplified neuroblastoma, ovarian cancer, triple‐negative breast cancer and small‐cell lung cancer.^20^, ^28^, ^29^, ^30^ While THZ1 has exhibited preliminary promising efficacy against PDAC, its definitive effectiveness appears to vary across different cases of the disease.^20^

PDAC is characterized by abundant genetic mutations, with the most notable quartet being *KRAS*, *TP53*, *CDKN2A* and *SMAD4* mutations.^31^ PDAC also exhibits considerable genetic heterogeneity. *KRAS* is the most commonly mutated gene in PDAC, and its mutation could activate a series of downstream oncogene signalling, inducing cancer genesis, progression and metastasis. G12D (with a change from glycine to valine at position 12 of the KRAS protein) and G12V (with a change from glycine to aspartic acid at position 12 of the KRAS protein) are the prominent mutant subtypes of *KRAS*. Our previous molecular pathologic epidemiology study suggested that patients with PDAC carrying the *KRAS‐G12D* mutation showed a significantly poorer prognosis than cases with wildtype *KRAS* or other mutations.^32^ Notably, the efficacy of anticancer drugs directly targeting KRAS remains uncertain. In mutant *KRAS*‐driven PDAC cells, *CDK7* knockout appeared to be as effective as *KRAS* knockout.^33^ The inhibitory effect of CDK7 inhibitors on PDAC may be dependent on the specific *KRAS* mutant subtype.^33^

In this study, we investigated the discrepant efficacies of THZ1 on PDAC with different *KRAS* mutants and the underlying mechanisms. We first analysed the association of CDK7 expression with survival by different *KRAS* mutants (*G12V* vs. *G12D*) in a Chinese cohort of patients with PDAC. Subsequently, we investigated CDK7 expression in various PDAC cell lines, and the effects of *CDK7* knockout on apoptosis, viability and proliferation of PDAC cells. We further explored the influences of THZ1 on the apoptosis and cell cycle of PDAC cells with different *KRAS* mutants, and on tumour growth using both cell‐derived xenograft (CDX) and mini‐CDX animal models. Moreover, we assessed the influence of THZ1 on transcription and on the expression and phosphorylation of RNAPOLII, and investigated the number of H3K27ac‐binding SEs and the effects of THZ1 on SE activity in PDAC cells with different *KRAS* mutants. Lastly, we evaluated the association of CDK7 expression with the PI3K/AKT/mTOR signalling, and the effects of THZ1 on H3K27ac binding with *PIK3CA* and on the PI3K/AKT/mTOR signalling and its downstream markers. Our findings may offer novel valuable clues for precise PDAC management and important evidence for future targeted trial design.

## 2 MATERIALS AND METHODS

### 2.1 Patients

We enrolled a cohort of consecutive cases with the incident, pathologically confirmed, primary PDAC, which were resected between January 2016 and August 2018 at Chinese PLA General Hospital (Beijing, China). Inclusion criteria encompassed the availability of haematoxylin and eosin slides with invasive cancer components, complete clinicopathologic features and follow‐up information, and without a prior cancer history. We excluded cases if they did not have a formalin‐fixed paraffin‐embedded (FFPE) sample from the primary cancer. We also excluded patients who survived less than 3 months after surgery to minimize the influence of perioperative events on survival outcomes. Data on cases from The Cancer Genome Atlas (TCGA) database^34^ who were diagnosed from January 2010 to December 2013 were also retrieved for survival analysis.

We gathered detailed baseline information, encompassing gender, age, cancer pathologic TNM stage, numbers of examined and positive lymph nodes, presence of vascular, lymphatic and perineural invasions, tumour location, size and differentiation grade, resection type, margin status, neoadjuvant and adjuvant therapies, and follow‐up data (survival time and status) for each patient in the Chinese cohort. Re‐evaluation of all pathology samples was conducted by two independent pathologists. All cases were (re)staged following the TNM staging system (eighth edition) by the American Joint Committee on Cancer (AJCC)/Union for International Cancer Control (UICC). All samples were assigned with anonymous codes in accordance with the local ethics regulations as stipulated by the Helsinki Declaration. We obtained written informed consent from all cases, and the Ethics Committee of Chinese PLA General Hospital approved this investigation.

### 2.2 Immunohistochemistry and scoring

Postoperative specimens from patients with PDAC were collected, fixed using formalin, embedded using paraffin and sectioned into 4 μm per piece of whole pathology sections. These sections were then incubated at 60°C for 1–2 h. Following microwave antigen retrieval, we applied .3% H_2_O_2_ to block endogenous peroxidase, and protein blocking was performed for 20 min. Subsequently, primary (1:100 dilution) and secondary antibodies were added sequentially, followed by incubation (2 and .5 h, respectively, at room temperature) and HRP labelling (40 min at room temperature). Diaminobenzidine solution (100 μL per slide) was then added (15 min), succeeded by haematoxylin background staining for 30 s. Gradient dehydration was performed using 80%, 95% and 100% ethanol, as well as 100% xylene.

Immunohistochemistry findings were independently assessed by two pathologists. CDK7 expression level was scored according to scores for both positive cell number (0, 0%–4%; 1, 5%–25%; 2, 26%–50%; 3, 51%–75%; 4, 76%–100%) and staining intensity (0–3 for negative, weak, moderate and strong). Score for positive cell number was then multiplied by the staining intensity score, generating a comprehensive score ranging from 0 to 12. Patients were subsequently categorized into two subgroups (low‐ and high‐expression subgroups) according to the median of the comprehensive CDK7 expression score.

### 2.3 DNA extraction, library preparation and next‐generation sequencing

As previously outlined,^32^ we extracted genomic DNAs from FFPE tumour specimens and matched adjacent control specimens with the QIAamp FFPE Tissue DNA Extraction Kit (Qiagen), and quantified them with the dsDNA HS Assay Kit (Thermo Fisher Scientific) using Qubit 3.0. We prepared DNA libraries with the KAPA HyperPrep Kit (Roche) and enriched them with the xGen Exome Research Panel (Integrated DNA Technologies), along with a Hybridization and Wash Kit. We then sequenced libraries for *KRAS* hotspot mutations with PE150 sequencing chemistry (Illumina) using the Illumina HiSeq4000 platform.

### 2.4 Single nucleotide variant

Processing of the sequencing data followed previously established procedures.^35^ For quality control, we used Trimmomatic to eliminate N bases or samples of low quality (with quality reading smaller than 20) from the FASTQ files. We used the Burrows‐Wheeler Aligner to map sequencing reads to hg19 (reference human genome). We utilized Picard to remove duplicate reads, followed by realignment around known insertions/deletions. We utilized GATK4 for base quality recalibration, and excluded samples with contamination rates exceeding .02 or Total Q Scores below 35. We used VCF2LR to eliminate non‐matching specimens from matched cancer–normal pairs, and used ContEst to evaluate cross‐sample contamination. We used Mutect to perform somatic SNV calling.^36^ We excluded SNVs with frequencies greater than 1% in the dbSNPs and 1000 Genomes Project. The SNVs underwent further filtration as described previously.^35^

### 2.5 Cell lines and reagents

We utilized patient‐derived and long‐established PDAC cell lines, encompassing those with *KRAS‐G12V* mutation (CAPAN2 and PANC03.27) and those with *KRAS‐G12D* mutation (SW1990, PANC02.03 and PANC10.05). We purchased these cell lines from the American Type Culture Collection (ATCC). SW1990 cells were cultivated in Leibovitz's L‐15 Medium with 10% foetal bovine serum (FBS). CAPAN2 cells were cultivated in McCoy's 5a Modified Medium with 10% FBS. PANC02.03, PANC03.27 and PANC10.05 cells were cultivated in RPMI‐1640 Medium with 15% FBS and 10 units/mL human recombinant insulin. SW1990 cells were cultivated at 37°C in 100% air, and the other cell lines were cultured at 37°C with 5% CO_2_ in a humidified atmosphere. *KRAS* mutation statuses of these cell lines were previously characterized.^37^, ^38^ Routine testing for mycoplasma was conducted using the Lonza MycoAlert (LT07‐418) mycoplasma detection kit, and all cell lines underwent repeated Short Tandem Repeat profiling for authentication. THZ1 was generously provided by the Gray Lab.^22^ Sources for the other reagents are detailed in Supplementary Methods.

### 2.6 CRISPR/CAS9 gene editing

The CRISPR/CAS9 technique was used to knock out *CDK7*. To prepare *CDK7*‐targeting pLKO‐tet‐on‐shRNAs, we synthesized oligonucleotides. After annealing, double‐stranded oligonucleotides were automatically linked to the tet‐on‐pLKO carrier (Addgene)^39^; sequences were: Scramble, forward: CGGGTGGACTCTTGAAAGTACTATCTCGAGATAGTACTTTCAAGAGTCCACTTTTTG, reverse: AATTAAAAAGTGGACTCTTGAAAGTACTATCTCGAGATAGTACTTTCAAGAGTCCAC; CDK7_1, forward: CCGGGCTGACACCATCACACATCAACTCGAGTTGATGTGTGATGGTGTCAGCTTTTTG, reverse: AATTCAAAAAGCTGACACCATCACACATCAACTCGAGTTGATGTGTGATGGTGTCAGC; CDK7_2, forward: CCGGGCTGTAGAAGTGAGTTTGTAACTCGAGTTACAAACTCACTTCTACAGCTTTTTG, reverse: AATTCAAAAAGCTGTAGAAGTGAGTTTGTAACTCGAGTTACAAACTCACTTCTACAGC. The construction of the lenti‐CRISPR/CAS9 vector adhered to the experimental steps of the backbone vector (Addgene).^40^ We selected the following sequences from public libraries^40^, ^41^: Scramble/GFP, forward: CACCGGGGCGAGGAGCTGTTCACCG, reverse: AAACCGGTGAACAGCTCCTCGCCCC; CDK7_1, forward: CACCGGAAGCTGGACTTCCTTGGGG, reverse: AAACCCCCAAGGAAGTCCAGCTTCC; CDK7_2, forward: CACCGATCTCTGGCCTTGTAAACGG, reverse: AAACCCGTTTACAAGGCCAGAGATC.

Lentiviruses were generated in HEK293T cells, which were cultured in DMEM with 10% FBS. Cells were transfected with packaging DNA plus tet‐on‐pLKO or lenti‐CRISPR vectors.^28^ Two micrograms vector DNA, 1.5 μg pCMV dR8.91, .5 μg pMD2‐VSVG and 12 μL lipid of Metafectene Pro (Biontex Laboratories GmbH) were utilized. DNA and lipid were prediluted individually in 300 μL of phosphate buffer saline (PBS) and then mixed. After incubation for 15 min, the DNA‐lipid mixtures were added to HEK293T cells. The viral supernatant was collected on the second and third days post‐transfection, filtered through .45‐μm membranes and added to target PDAC cells in the presence of 8 μg/mL polybrene (Millipore). Cells were treated with 1.5 μg/mL puromycin for 2 days for selection.

### 2.7 Cell proliferation assay

We assessed cell proliferation utilizing the clonogenic assay. Cells transfected with the lenti‐CRISPR vector were collected and seeded into 24‐well plates (1000 cells per well) following puromycin selection. The cells were then cultured for 7−10 days. After crystal violet staining, images were captured through scanning.

### 2.8 Cell apoptosis assay

Apoptosis was evaluated by measuring the Caspase 3/7 activity. On day 1, we seeded cells into a 96‐well plate (around 1×10^4^ cells per well). On day 2, we treated cells with THZ1, at gradient concentrations of 0 (dimethyl sulfoxide [DMSO]), 50, 100 and 200 nM. Each experiment was repeated thrice. Cell apoptosis was evaluated at 24 and 48 h, by adding Caspase 3/7 reagent and culture medium (1:1) to the 96‐well plate (100 μL per well). The plate was then covered with tin foil and placed in an incubator for 2 h. Subsequently, we measured the fluorescence intensity of each well.

### 2.9 Cell cycle assay

For cell cycle evaluation, flow cytometry was employed. We placed cells into 6‐well plates and treated them with THZ1 (0, 100 and 200 nM) for 1 and 2 days. After treatment, cells were trypsinized, centrifuged, fixed using 70% precooled (−20°C) ethanol to prepare the single‐cell suspension, washed and exposed to PI/RNase staining buffer at room temperature for 15 min. The cells were then passed through a 300‐mesh screen into a flow cytometry tube, which was placed in an ice box. Cells were examined using a flow cytometer, and the obtained images were analysed using Flowjo software, with the removal of cell fragments and multiple cell connections.

### 2.10 Western blot

For the extraction of total cell protein, the radioimmunoprecipitation assay buffer (including lysis buffer, phosphatase inhibitor and proteinase inhibitor; Life Technologies) was utilized. A protein sample of 30 μg was loaded for gel electrophoresis, which lasted for 1.5 h. The proteins were subsequently transferred to the PVDF membrane (Bio‐Rad) overnight, followed by 1‐h blocking with 5% skimmed milk. The membrane was incubated with primary antibody overnight at 4°C, and then with secondary antibody at room temperature for 1 h. Images were acquired with an Odyssey Infrared scanner.

### 2.11 Chromatin immunoprecipitation (ChIP) sequencing (ChIP‐seq) and SE identification

ChIP with anti‐H3K27ac antibody as the target antibody and IgG as the negative control was performed using the Magna ChIP Kit (Millipore) according to the manufacturer's instructions.^42^ The reagents included H3K9me3 antibody and SAT primer, which were used as intra‐group control to validate experiment accuracy. Details on ChIP are provided in Supplementary Methods.

We sequenced ChIP‐seq libraries using the HiSeq 2000 platform provided by Novogene. SEs were identified utilizing the Rank Ordering of Super‐Enhancers (ROSE) algorithm. SEs had two or more H3K27ac peaks (detected using MACS2) within a range of 12.0 kb, and were situated more than 2.5 kb from the transcription start site. SEs were further defined as those with the highest level of H3K27 acetylation, via graphing the inflection plot and selecting values where the slope of the fitted curve exceeded 1. Enhancers that were below the point on the curve with the slope of 1 were categorized as typical‐enhancers (TEs).^8^

### 2.12 RNA‐sequencing

We isolated total RNA using the TRIzol (Invitrogen) reagent. The cDNA library was constructed by Novogene. We performed sequencing on the Illumina HiSeq 2500 platform (Novogene). Transcription quantification and differential expression analyses were conducted with the DESeq package of R software, with a cutoff *p* < .05. Gene Ontology (GO) enrichment analysis was performed using the clusterProfiler package of R software.

### 2.13 PDAC CDX model

Stable overexpression of *firefly luciferase* (*Fluc*) was achieved in the tumour cell lines. A lentiviral vector encoding the humanized *Fluc* gene (GeneChem) was used to transduce the parental tumour cell lines according to the manufacturer's instructions. Tumour cells were incubated with viral stocks supplemented with 4 μg/mL Polybrene (Sigma‐Aldrich) for 6 h, and then replenished with fresh medium. The transduced cells were exposed to 2 μg/mL puromycin (Sigma‐Aldrich) 2 days post‐transduction to eliminate non‐transduced cells. Engineered *Fluc*‐positive tumour cell clones were obtained 10 days after lentivector transduction. Subsequently, we subcutaneously injected 1×10^7^ engineered CAPAN2 or SW1990 cells in 150 μL of PBS into the left flank near the posterior limb of 6‐week‐old BALB/c nude mice weighing 20–25 g. When the tumour volume reached 50−100 mm^3^, we randomly divided mice with each cell type into three groups, with six mice in each group. These groups were treated intraperitoneally with PBS, 5 mg/kg THZ1 or 10 mg/kg THZ1 twice daily on days 3 and 6 every week for 3 weeks. Tumour growth and anti‐tumour efficacy were assessed using the in vivo Imaging System (IVIS; PerkinElmer) after intravenous injection of 150 mg/kg D‐Luciferin solution into the mice. The mice were euthanized 3 weeks after implantation.

### 2.14 Mini‐CDX model

The OncoVee Mini‐Patient Derived Xenograft (PDX) kit (LIDE Biotech) was also employed to evaluate drug efficacy. Briefly, cells were collected and transferred to Hank's Balanced Salt Solution‐washed capsules, which were subcutaneously implanted into 5‐week‐old BALB/c nude mice through a small skin incision, where each mouse received three capsules. Six paired capsules were assessed. THZ1 (10 mg/kg, IP, BID) was administered for 7 days. Finally, we removed the capsules, and evaluated tumour cell proliferation and viability according to relative fluorescence units (RFUs) with the CellTiter‐Glo Luminescent Cell Viability Assay (Promega). We computed the relative proliferation rate of tumour cells (T/C (%)) as the percentage ratio of the relative proliferation of the intervention group compared to the control group: T/C (%) = cell viability of the intervention group/cell viability of the control group×100%. The lower the value, the more potent the drug inhibition on tumour cells. We compared results between the vehicle and THZ1 groups using the paired‐sample *t*‐test.

The Institutional Review Board of Chinese PLA General Hospital approved the animal experiments, which were performed according to the Guidelines for the Care and Use of Laboratory Animals by the NIH.

### 2.15 Statistics

Continuous data were shown as mean ± standard deviation (or standard error where appropriate) and median (interquartile range), while categorical data were shown as count (percentage). We compared continuous data between groups using *t*‐test, ANOVA (with *post hoc* analysis), Wilcoxon or Kruskal–Wallis test as appropriate. Categorical variables were compared utilizing *χ* ^2^ or Fisher's exact test where appropriate unless otherwise specified. We evaluated correlations between two continuous variables using Pearson or Spearman correlation analysis where appropriate. Prognosis endpoints were disease‐free survival (DFS) and OS. DFS was defined as the time to cancer recurrence at any site or death due to any cause, whichever first occurred, and OS was the time to all‐cause death. Univariable survival was computed utilizing the Kaplan−Meier method and compared between groups utilizing the log‐rank test. Association of CDK7 expression with survival was further assessed utilizing multivariable‐adjusted Cox proportional hazards regression. The TCGA transcriptome sequencing database was also utilized to analyse the association of *CDK7* mRNA expression with PDAC prognosis, using the online tools (http://ualcan.path.uab.edu/ and www.oncolnc.org). We performed statistical analyses using R 4.2.0 and GraphPad Prism 9.4.1 software. Statistical significance was indicated by two‐sided *p* < .05.

## 3 RESULTS

### 3.1 CDK7 expression was inversely associated with prognosis of patients with PDAC

**FIGURE 1:**
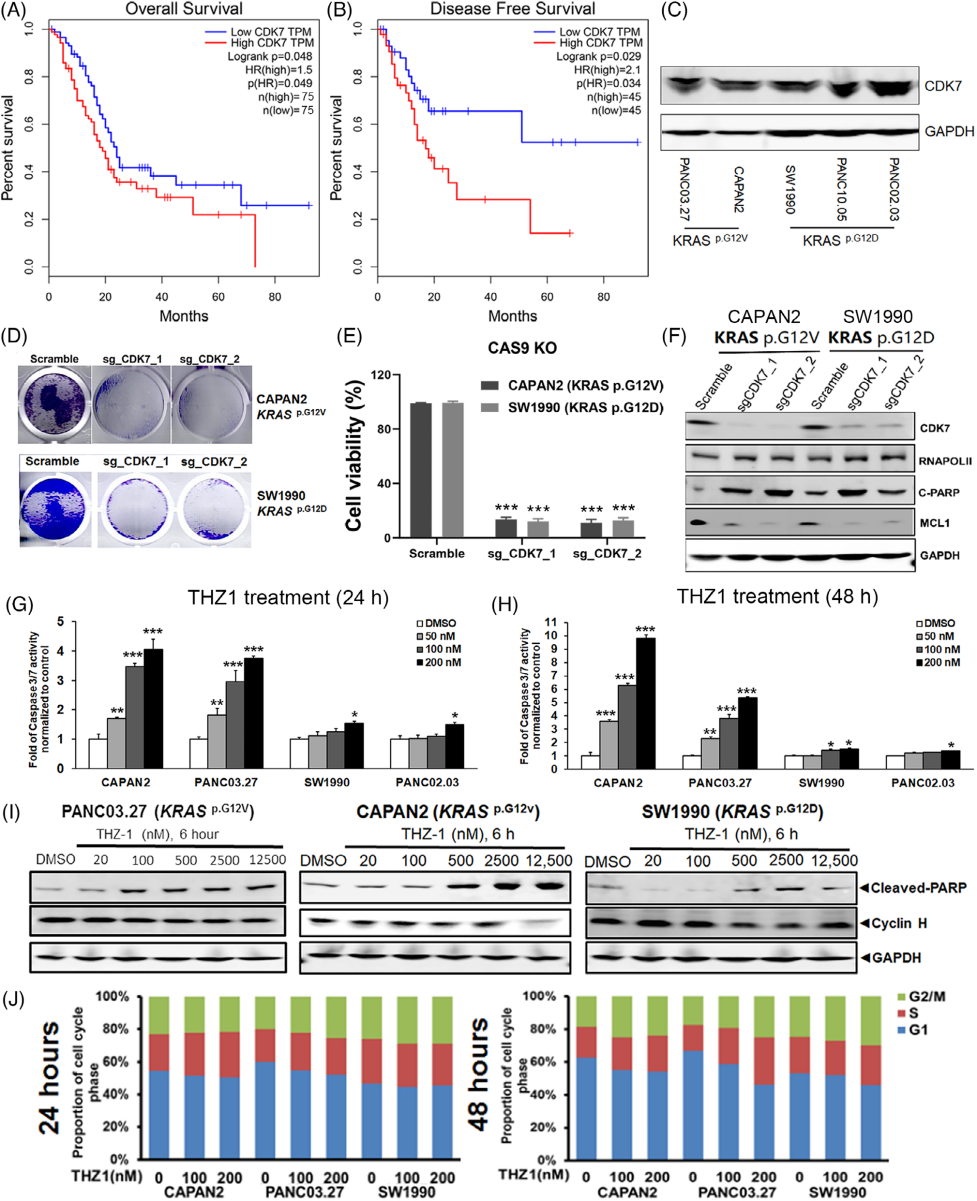
CDK7 was essential for the viability and proliferation of pancreatic ductal adenocarcinoma (PDAC) cells, and the suppressive effect of the CDK7 inhibitor THZ1 on PDAC was dependent on *KRAS* mutation subtypes in vitro. (A, B) A high expression of *CDK7* was significantly associated with poorer overall survival (A) and disease‐free survival (B) in the PDAC cohort of The Cancer Genome Atlas database. (C) Expression of CDK7 in PDAC cell lines with different *KRAS* mutations by Western blot. (D, E) In CAPAN2 (*KRAS‐G12V*) and SW1990 (*KRAS‐G12D*) cell lines, cell proliferation/viability was significantly weakened after CRISPR/CAS9‐mediated *CDK7* knockout. (F) Transcription‐ and apoptosis‐associated protein level changes after *CDK7* knockout in the CAPAN2 and SW1990 PDAC cell lines using the CRISPR/CAS9 technology. (G, H) Apoptosis of PDAC cell lines with different *KRAS* mutations after THZ1 treatment (0, 50, 100 and 200 nM) for 24 h (G) and 48 h (H), respectively. (I) Effects of THZ1 of different concentrations on the levels of cleaved‐PARP and Cyclin H in PDAC cell lines with different *KRAS* mutations (PANC03.27, CAPAN2 and SW1990). (J) Cell cycle of PDAC cell lines with different *KRAS* mutations after THZ1 treatment (0, 100 and 200 nM) for 24 and 48 h, respectively. In E, G and H, the bars suggest the standard errors of the mean values after three repeated experiments. *p < .05; **p < .01; ***p < .001.

Utilizing the entire genome expression data from the PDAC cohort within the TCGA transcriptome sequencing database, we dichotomized cases into *CDK7*‐high and *CDK7*‐low expression groups, employing the median *CDK7* mRNA expression level as the cutoff. Cases with high *CDK7* expression had significantly worse OS (hazard ratio [HR] = 1.5, *p* = .049; available in 150 patients; Figure 1A) and worse DFS (HR = 2.1; *p* = .034; available in 90 patients; Figure 1B).

### 3.2 Association between CDK7 expression and survival depended on the KRAS mutant subtype in PDAC

In the PDAC cohort from Chinese PLA General Hospital, 241 patients with resected PDAC and any *KRAS* mutation (median age, 67 years; male proportion, 56%) were examined (Table S3). Among them, 100 patients (41%) had the *KRAS‐G12D* mutation, and 80 (33%) carried the *KRAS‐G12V* mutation. Significant differences in the analysed patient, tumour or treatment characteristics between patients with the two distinct *KRAS* mutation subtypes were not observed, except for a higher prevalence of lymphatic invasion within the *KRAS‐G12D* group. No significant differences in CDK7 expression scores were evident between the two groups (*p* = .506). Compared with patients having the *KRAS‐G12V* mutation, those having the *KRAS‐G12D* mutation had notably shorter median OS (14 vs. 24 months, *p* < .001) and DFS (10 vs. 17 months, *p* = .003).

After excluding patients who died within 3 months following resection (Table 1), in the *KRAS‐G12D* group, the expression of CDK7 (localized within the nucleus) based on immunohistochemistry findings was not significantly linked to either OS or DFS. However, in the *KRAS‐G12V* group, low CDK7 expression was significantly linked to improved DFS (HR = .32, *p* = .035) and tended to be linked to enhanced OS (HR = .41, *p* = .083), with adjustment for other potential prognostic factors.

**TABLE 1:** Multivariable‐adjusted associations of CDK7 expression with overall survival (OS) and disease‐free survival (DFS) after excluding cases who died within 3 months after resection in our resected pancreatic cancer cohort, stratified by *KRAS*.

	*KRAS G12D*		*KRAS G12V*	
Variables	Adjusted HR (95% CI)	*p*	Adjusted HR (95% CI)	*p*
*OS*				
CDK7 high	1.00 (ref.)		1.00 (ref.)	
CDK7 low	.66 (.34−1.26)	.207	.41 (.15−1.13)	.083
*DFS*				
CDK7 high	1.00 (ref.)		1.00 (ref.)	
CDK7 low	.62 (.32−1.21)	.160	.32 (.11−.92)	**.035**

*Note*: Hazard ratios (HRs) and corresponding 95% confidence intervals (CIs) for associations of low versus high CDK7 expression with OS and DFS were calculated using multivariable Cox proportional hazards regression adjusting for age, sex, tumour location, size, pT stage, pN stage, differentiation grade, vascular invasion, lymphatic invasion, perineural invasion, resection type, resection margin, number of dissected lymph nodes, neoadjuvant therapy and adjuvant therapy. The bold *p* value indicates statistical significance (*p* < 0.05).

### 3.3 The viability of PaC cells with different KRAS mutations highly depended on CDK7

The expressions of CDK7 in PDAC cells with distinct *KRAS* mutations (*KRAS‐G12V*: PANC03.27 and CAPAN2; *KRAS‐G12D*: SW1990, PANC10.05 and PANC02.03) are shown in Figure 1C, and both *KRAS* mutation subtypes exhibited abundant CDK7 expression, suggesting CDK7 as a potential therapeutic target. PDACs exhibit a significant dependency on CDK7‐mediated transcription.^20^ Using two distinct sg‐*CDK7s* (sg‐*CDK7*‐1 and sg‐*CDK7*‐2) for each cell type, CRISPR/CAS9‐mediated *CDK7* knockout, which resulted in virtually no CDK7 protein expression, markedly and significantly hindered cell proliferation and viability (Figure 1D,E). *CDK7* knockout concurrently induced apoptosis as evidenced by substantially elevated levels of the apoptosis‐associated protein cleaved‐PARP and noticeably reduced levels of the anti‐apoptosis protein MCL1 (Figure 1F) across both PDAC cell types, featuring *KRAS‐G12V* (CAPAN2) and *KRAS‐G12D* (SW1990) mutations. *CDK7* knockout did not notably alter the expression of RNAPOLII in either cell line (Figure 1F). CDK7 knockout markedly decreased RNAPOLII CTD phosphorylation at ser2, ser5 and ser7 in both *KRAS‐G12V* and *KRAS‐G12D* mutant PDAC cells, with the inhibitory effects appearing more potent in *KRAS‐G12V* cells, particularly for the phosphorylation at ser5 (Figure S1). Complete inhibition of RNAPOLII CTD phosphorylation was not achieved post CDK7 knockout, possibly due to the presence of other CDKs, including CDK12 and CDK13 which are also susceptible to inhibition by THZ1.

### 3.4 THZ1 selectively suppressed PDAC based on the different KRAS mutant subtypes in vitro

After a 72‐h treatment period, THZ1 exhibited more robust inhibitory effects on viability in CAPAN2 (IC_50_ = .025 μM) and PANC03.27 (IC_50_ = .056 μM) cells carrying *KRAS‐G12V* mutations compared with SW1990 (IC_50_ = .195 μM), PANC10.05 (IC_50_ = .093 μM) and PANC02.03 (IC_50_ = .103 μM) cells carrying *KRAS‐G12D* mutations (Figure S2A). THZ1 significantly induced apoptosis in both a time‐dependent and dose‐dependent manner in PDAC cells with *KRAS‐G12V* mutations (CAPAN2 and PANC03.27), as measured by Caspase 3/7 activity (50 nM THZ1 already significantly induced apoptosis). This apoptosis induction was considerably more pronounced than in cells with *KRAS‐G12D* mutations (SW1990 and PANC02.03; Figure 1G,H). Correspondingly, THZ1 treatment (0–12 500 nM) for 6 h significantly elevated the level of cleaved‐PARP, an apoptosis marker, in a dose‐dependent manner in PDAC cells. This effect was more potent in cells with *KRAS‐G12V* mutations (PANC03.27 and CAPAN2) compared with cells with *KRAS‐G12D* mutations (SW1990; Figure 1I). The effect of THZ1 on Cyclin H expression was minor in both *KRAS* mutation subtypes, except for a significant reduction in Cyclin H expression in CAPAN2 (*KRAS‐G12V*) cells treated with 12 500 nM THZ1 (Figure 1I). Accordingly, THZ1 treatment had an insignificant effect on the cell cycle as indicated by the changes in the proportions of G2/M, S and G1 phases in both *KRAS‐G12V* (CAPAN2 and PANC03.27) and *KRAS‐G12D* (SW1990) PDAC cells (Figure 1J).

YKL‐5‐124, a covalent CDK7 inhibitor, exhibits cellular and biochemical selectivity for CDK7 over CDK12/13. YKL‐5‐124 causes robust cell cycle arrest while exerting a surprisingly weak effect on RNAPOLII phosphorylation.^43^ The inhibitory effect of YKL‐5‐124 on cell viability and proliferation was strongest in PANC03.27 (*KRAS‐G12V*) and PANC02.03 (*KRAS‐G12D*) cells, but was less pronounced in CAPAN2 (*KRAS‐G12V*) cells than in PANC10.05 (*KRAS‐G12D*) cells (Figure S2B). This finding suggested that the inhibitory effect of YKL‐5‐124, which is largely different from THZ1, was not *KRAS* mutation‐dependent.

### 3.5 THZ1 selectively suppressed PDAC based on the different KRAS mutation subtypes in vivo

**FIGURE 2:**
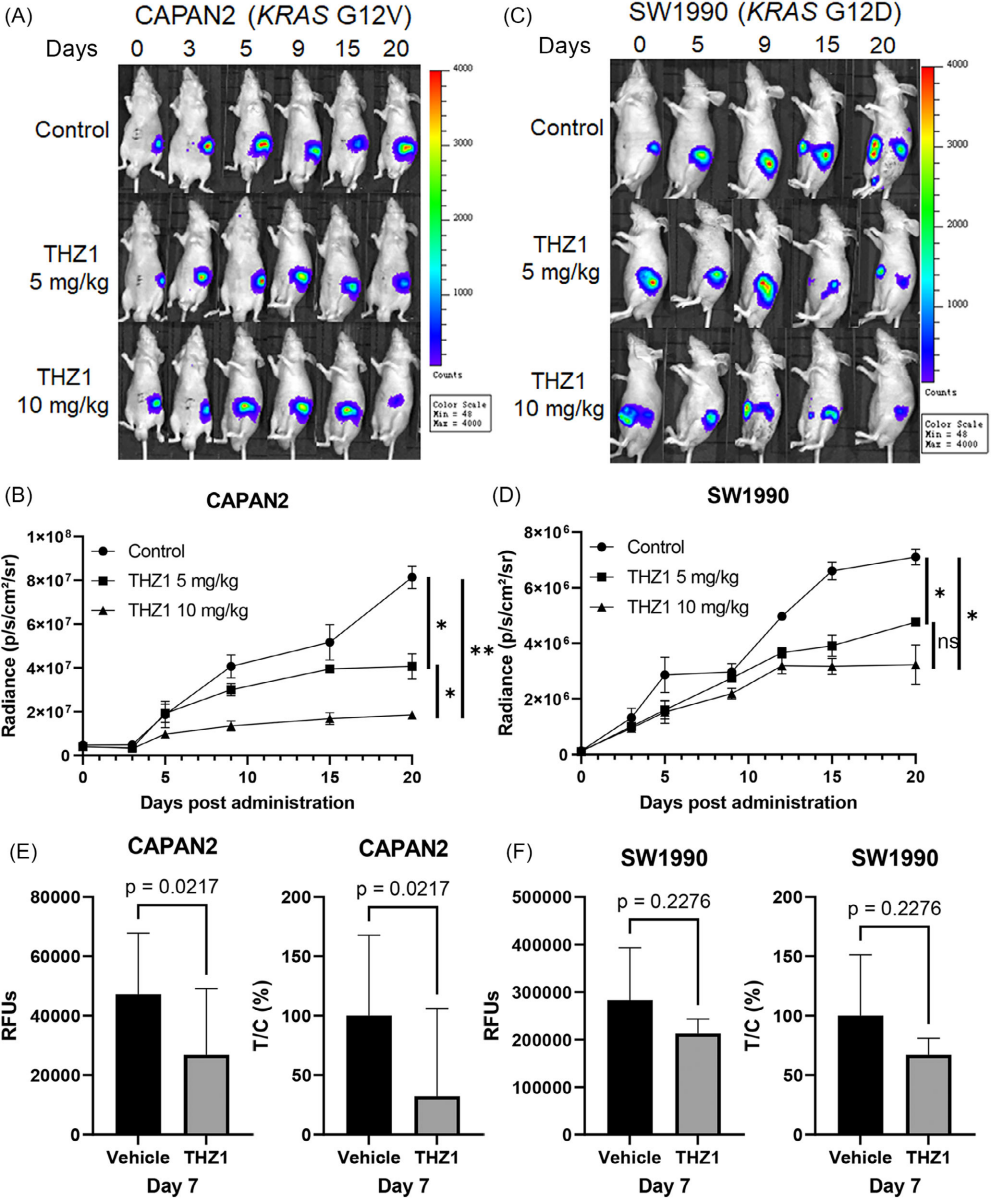
The suppressive effect of the CDK7 inhibitor THZ1 on cell‐derived xenograft (CDX) and mini‐CDX models of pancreatic ductal adenocarcinoma (PDAC) depended on *KRAS* mutation subtypes in vivo. (A–D) CDX models for the effects of 5 and 10 mg/kg THZ1 on the growth of tumour originating from CAPAN2 (*KRAS‐G12V*; A, B) and SW1990 (*KRAS‐G12D*; C, D) cells. (E, F) Mini‐CDX models revealed that THZ1 selectively suppressed PDAC based on the *KRAS* mutation subtype, as reflected by relative fluorescence units (RFUs) and relative proliferation rates (T/C (%)) of tumour cells (E, CAPAN2; F, SW1990) treated with vehicle control and THZ1 (10 mg/kg, IP, BID). *p < .05.

THZ1 (5 and 10 mg/kg, twice per day) significantly hindered the growth of xenograft PDAC tumours in mice derived from CAPAN2 cells with *KRAS‐G12V* mutation in a dose‐dependent manner (Figure 2A,B). THZ1 administration at both 5 and 10 mg/kg also significantly inhibited the growth of xenograft tumours originating from SW1990 cells with *KRAS‐G12D* mutation, but no significant discrepancy in the inhibitory effects was noted between the two dose groups (Figure 2C,D). On day 20 post THZ1 treatment, the relative proliferation rates of cancer cells T/Cs (%) were significantly lower in CAPAN2 (*KRAS‐G12V*) cells than in SW1990 (*KRAS‐G12D*) cells with both 5 mg/kg (50.0% vs. 67.0%, *p* = .047) and 10 mg/kg (22.7% vs. 45.5%, *p* = .013) THZ1.

In mini‐CDX models, 10 mg/kg THZ1 distinctly suppressed CAPAN2 (*KRAS‐G12V*) cancer cells, evident through both significantly reduced RFU and T/C (%) values compared with the vehicle control group on day 7 (*p* = .022; Figure 2E). However, the suppressive effect of 10 mg/kg THZ1 on tumour growth was not significant in mini‐CDX models implanted with SW1990 (*KRAS‐G12D*) cells (*p* = .228; Figure 2F).

### 3.6 THZ1 significantly reduced the transcription level of PDAC cells and selectively inhibited the phosphorylation of RNAPOLII CTD in PDAC cells according to different KRAS mutation subtypes

**FIGURE 3:**
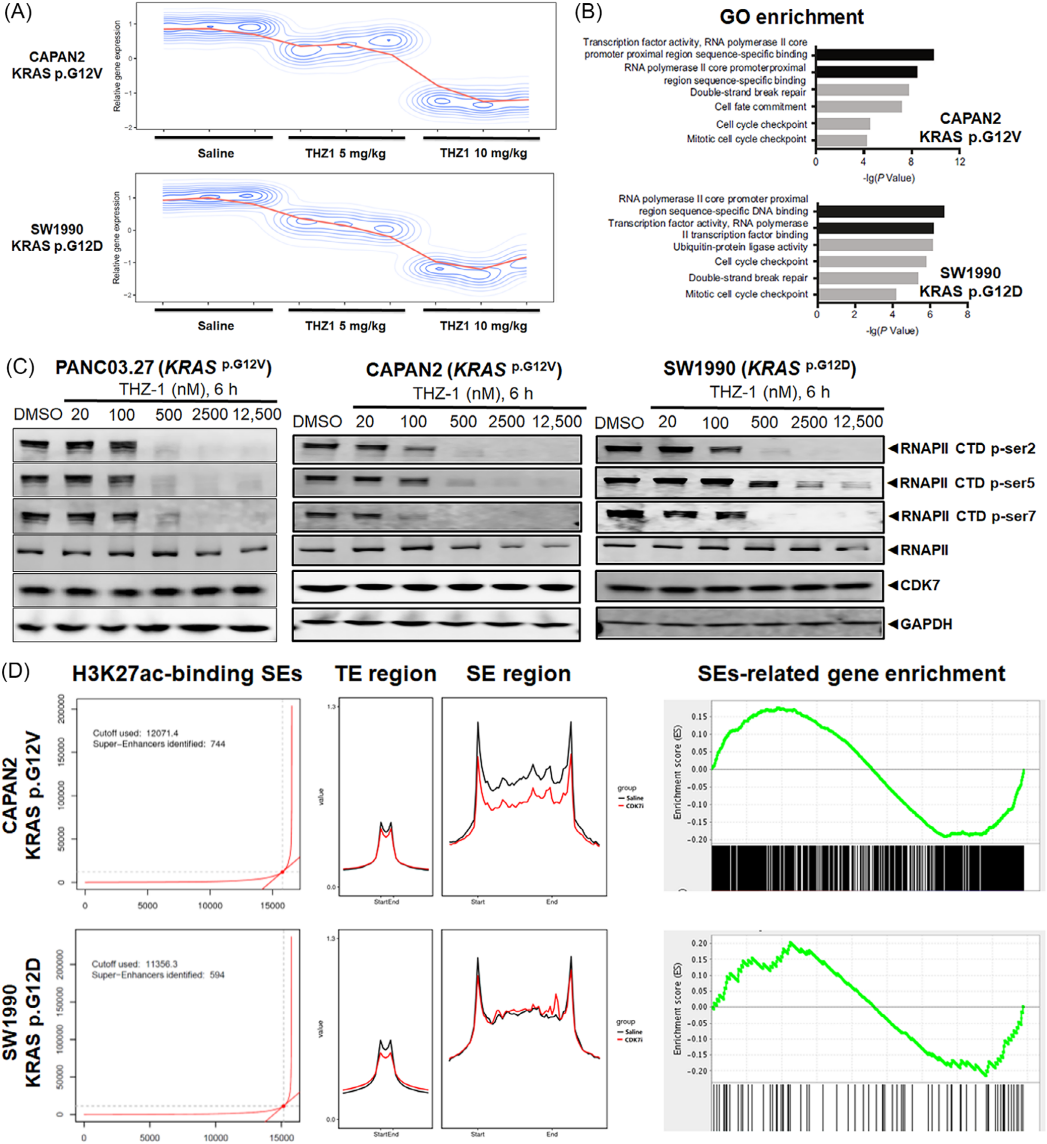
THZ1 suppressed the global transcription in pancreatic ductal adenocarcinoma (PDAC), which was associated with the RNA polymerase II (RNAPOLII) activity, and THZ1 selectively inhibited the expression and phosphorylation of RNAPOLII and super‐enhancer (SE) activity accordingly to *KRAS* mutation subtypes. (A, B) THZ1 (5 and 10 mg/kg) inhibited the global transcription in PDAC in a dose‐dependent manner (A), which was associated with the RNAPOLII activity (B). (C) The inhibitory effect of treatment with THZ1 of different concentrations (20, 100, 500, 2500 and 12 500 nM) or control (DMSO) for 6 h on the expression and phosphorylation of the CTD of the transcription‐related protein RNAPOLII in PDAC cell lines with different *KRAS* mutations (PANC03.27, CAPAN2 and SW1990). (D) Number of H3K27ac‐binding super‐enhancers, enrichment of super‐enhancer‐related genes and effects of THZ1 on super‐enhancer activity in CAPAN2 (*KRAS‐G12V*) and SW1990 (*KRAS‐G12D*) cells.

THZ1 markedly diminished global gene transcription levels in a dose‐dependent manner in both PDAC cell types, featuring *KRAS‐G12V* (CAPAN2) and *KRAS‐G12D* (SW1990) mutations, as indicated by RNA‐sequencing (RNA‐seq) data (Figure 3A). GO enrichment analyses based on our RNA‐seq data revealed that *KRAS‐G12V* (CAPAN2) was most prominently linked to ‘transcription factor activity, RNAPOLII core promoter proximal region sequence‐specific binding’, and that *KRAS*‐*G12D* (SW1990) was most strongly associated with ‘RNAPOLII core promoter proximal region sequence‐specific DNA binding’ and ‘transcription factor activity, RNAPOLII transcription factor binding’ (Figures 3B and S3).

THZ1 treatment (20–12 500 nM) for 6 h notably curbed the levels of RNAPOLII CTD phosphorylation at ser2, ser5 and ser7 in a dose‐dependent manner, suggesting transcription inhibition. This suppressive activity was stronger in PDAC cells with *KRAS‐G12V* mutation (PANC03.27 and CAPAN2, particularly CAPAN2) than in cells with *KRAS‐G12D* mutation (SW1990 and PANC02.03) at equivalent doses, with a noticeable emphasis on phosphorylation at ser5 (Figure 3C). At a concentration of 500 nM, THZ1 nearly completely halted phosphorylation at ser2, ser5 and ser7 in *KRAS‐G12V* cells. The total expression of RNAPOLII was also slightly more profoundly suppressed by THZ1 in *KRAS‐G12V* cells than in *KRAS‐G12D* cells, while CDK7 expression was not significantly influenced by THZ1 (Figures 3C and S3). THZ1 also significantly suppressed RNAPOLII phosphorylation in a time‐dependent manner, with the inhibitory effect being also more potent in PDAC cells with *KRAS‐G12V* mutation (PANC03.27 and CAPAN2) than in cells with *KRAS‐G12D* mutation (SW1990; Figure S4).

### 3.7 THZ1 selectively inhibited the SE activity in PDAC cells with KRAS‐G12V mutation but not in cells with KRAS‐G12D mutation

Through ChIP‐seq analysis, a total of 744 and 594 H3K27ac‐binding SEs were identified in PDAC cells with *KRAS‐G12V* (CAPAN2) and *KRAS‐G12D* (SW1990) mutants, respectively (Figure 3D). CAPAN2 cells (*KRAS‐G12V*) also exhibited a more robust enrichment of SE‐related genes than SW1990 cells (*KRAS‐G12D*). When compared with the saline control, THZ1 significantly inhibited the SE region activity in CAPAN2 cells carrying *KRAS‐G12V* mutation, while such an effect was not evident in SW1990 cells carrying *KRAS‐G12D* mutation. However, the TE region activity was more noticeably diminished in SW1990 (*KRAS‐G12D*) cells compared with CAPAN2 (*KRAS‐G12V*) cells.

### 3.8 CDK7 expression was positively linked to the PI3K/AKT/mTOR signalling in PDAC

**FIGURE 4:**
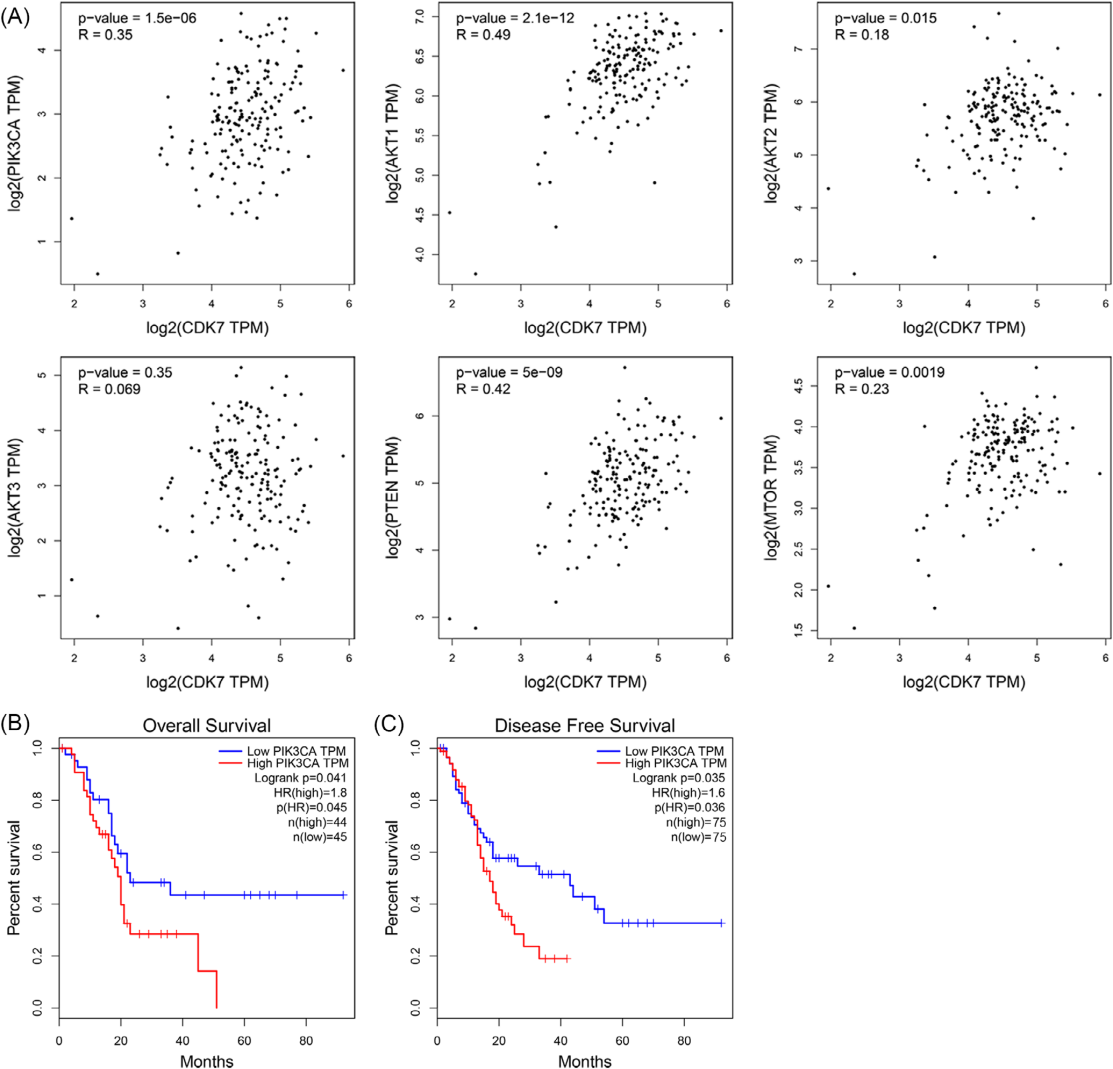
The expression of *CDK7* was positively associated with the PI3K/AKT/mTOR signalling in pancreatic ductal adenocarcinoma (PDAC). (A) Associations of the level of *CDK7* with the levels of *PIK3CA*, *AKT1/2/3*, *MTOR* and *PTEN*. (B, C) Associations of the expression of *PIK3CA* with overall survival (B) and disease‐free survival (C).

In PDAC, activation of the oncogenic PI3K/AKT/mTOR signalling is linked to increased tumour growth, drug resistance and epithelial‐mesenchymal transition, weakened apoptosis and tumour immunogenicity, and worse pathological and clinical outcomes.^44^, ^45^, ^46^, ^47^, ^48^ In the PDAC cohort from the TCGA database, we observed a significant positive association of *CDK7* mRNA expression with the mRNA expressions of *PIK3CA* (*R* = .35, *p* < .001), *AKT1* (*R* = .49, *p* < .001), *AKT2* (*R* = .18, *p* = .015), *PTEN* (*R* = .42, *p* < .001) and *mTOR* (*R* = .23, *p* = .002), which are all involved in the PI3K/AKT/mTOR signalling pathway, after log2 transformation (Figure 4A). Furthermore, patients having high *PIK3CA* expression had significantly worse OS (HR = 1.8, *p* = .045; Figure 4B) and poorer DFS (HR = 1.6; *p* = .036; Figure 4C).

### 3.9 THZ1 markedly reduced the PIK3CA binding level of H3K27ac, a marker for SE activity, in PDAC

**FIGURE 5:**
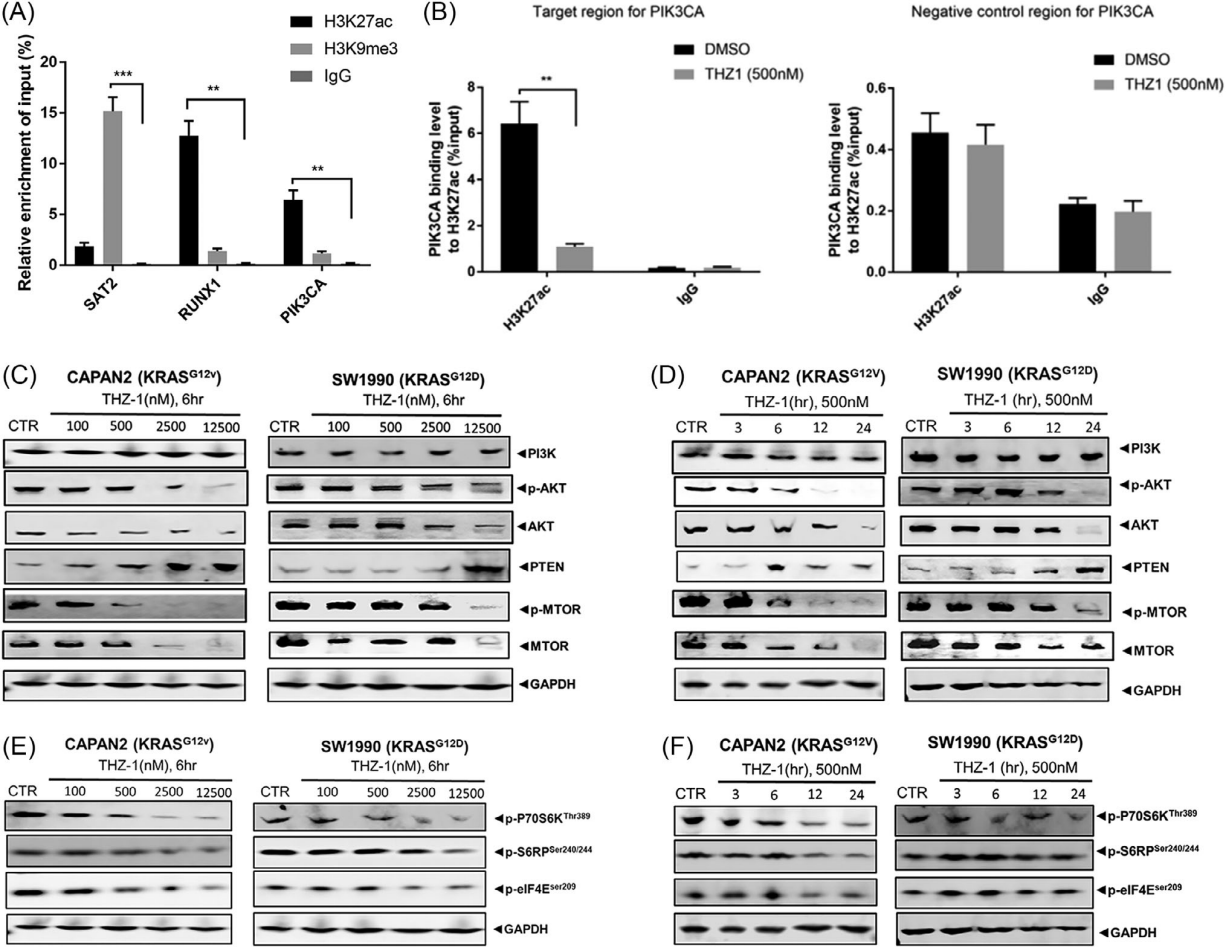
THZ1 inhibited the binding of H3K27ac to *PIK3CA*, and discrepantly suppressed the PI3K/AKT/mTOR signalling according to different *KRAS*‐mutant subtypes in pancreatic ducal adenocarcinoma (PDAC). (A) Relationship between H3K27ac and *PIK3CA* in CAPAN2 cells with *KRAS‐G12V* mutation in the ChIP experiment. (B) Effect of THZ1 on the binding of H3K27ac with *PIK3CA* in CAPAN2 cells using *PI3KCA*‐specific and negative control primers, respectively. (C, D) Effects of THZ1 on the expression of the PI3K/AKT/mTOR pathway‐related proteins in CAPAN2 (*KRAS‐G12V*) and SW1990 (*KRAS‐G12D*) cells by THZ1 concentration (0, 100, 500, 2500 and 12 500 nM; C) and treatment time (0, 3, 6, 12 and 24 h; D). (E, F) Effects of THZ1 on the phosphorylation of the downstream markers of mTOR (P70S6K, S6RP and eIF4E) in CAPAN2 and SW1990 cells by THZ1 concentration (0, 100, 500, 2500 and 12 500 nM; E) and treatment time (0, 3, 6, 12 and 24 h; F). The bars in A and B show the standard errors of the mean values after three repeated experiments. C and E shared the same set of controls, and E and F shared the same set of controls. *p < .05; **p < .01; ***p < .001.

In the ChIP experiments, the H3K27ac antibody was used as the target antibody. To verify the specificity of the ChIP experiments, we performed a double‐control verification on the DNA samples extracted utilizing the H3K27ac antibody, H3K9me3 antibody and IgG antibody. *SAT2* and *RUNX1* genes were selected as controls. As shown in Figure 5A, the H3K9me3 antibody displayed a high enrichment of *SAT2* but not *RUNX1*, while the H3K27ac antibody exhibited strong enrichment of *RUNX1* but not *SAT2*. These confirmed the good specificity of the H3K27ac antibody and the high accuracy of our experiment.

In CAPAN2 PDAC cells with *KRAS‐G12V* mutation, the level of *PIK3CA* in the DNA bound by H3K27ac antibody was significantly higher than in that bound by H3K9me3 antibody or IgG antibody, suggesting that histone H3K27 might regulate *PIK3CA* expression through acetylation (Figure 5A). Subsequently, CAPAN2 PDAC cells were treated with 500 nM THZ1 for 6 h, followed by a ChIP experiment using H3K27ac antibody. PCR was conducted on the obtained DNA using specific primers for *PI3KCA* and negative control (Figure 5B). After adding 500 nM THZ1, the level of *PIK3CA* in the DNA bound by H3K27ac antibody significantly decreased in CAPAN2 cells carrying *KRAS‐G12V* mutation, indicating a reduction in the binding of H3K27ac histone with *PIK3CA* (Figure 5B). However, the level of *PIK3CA* binding to H3K27ac in SW1990 cells with *KRAS‐G12D* mutation was not significantly reduced by 500 nM THZ1 (Figure S5).

The ChIP experiments revealed that *PIK3CA* expression was significantly regulated by H3K27ac compared to the controls (Figure 5A), and substantiated that THZ1 (500 nM) significantly weakened the binding of H3K27ac to *PIK3CA* in CAPAN2 (*KRAS‐G12V*) cells (Figure 5B), but not in SW1990 (*KRAS‐G12D*) cells (Figure S5).

### 3.10 THZ1 discrepantly suppressed the PI3K/AKT/mTOR signalling in PDAC cells of different KRAS‐mutant subtypes

THZ1 downregulated the expression of AKT and mTOR and lowered their phosphorylation levels (with a more pronounced effect on phosphorylation than on protein expression), while it upregulated the expression of PTEN. This was observed in both a dose‐dependent manner (6 h, 0–12 500 nM; Figure 5C) and a time‐dependent manner (500 nM, 0–24 h; Figure 5D) in PDAC cells. All of these effects were more pronounced in CAPAN2 cells with *KRAS‐G12V* mutation than in SW1990 cells with *KRAS*‐*G12D* mutation. THZ1 did not significantly influence the expression of the total PI3K protein, which includes four subtypes, with PI3KCA being the catalytic subunit of Type IA PI3K. Most current antibodies cannot precisely differentiate between each subtype; they can only mark the protein level of mixed PI3K.^49^ Additionally, THZ1 significantly inhibited the phosphorylation of downstream markers of mTOR, and downregulated the levels of p‐P70S6K^Thr389^, p‐S6RP^Ser240/244^ and p‐eIF4E^Ser209^. This occurred in both a dose‐dependent manner (6 h, 0–12 500 nM; Figure 5E) and a time‐dependent manner (500 nM, 0–24 h; Figure 5F) in PDAC cells. The inhibitory effects were also more pronounced in CAPAN2 cells with *KRAS‐G12V* mutation than in SW1990 cells with *KRAS*‐*G12D* mutation. Treated with 500 nM THZ1, the expression levels of the three phosphorylated markers exhibited only marginal changes from 0 to 24 h in SW1990 (*KRAS*‐*G12D*) cells.

## 4 DISCUSSION

**FIGURE 6:**
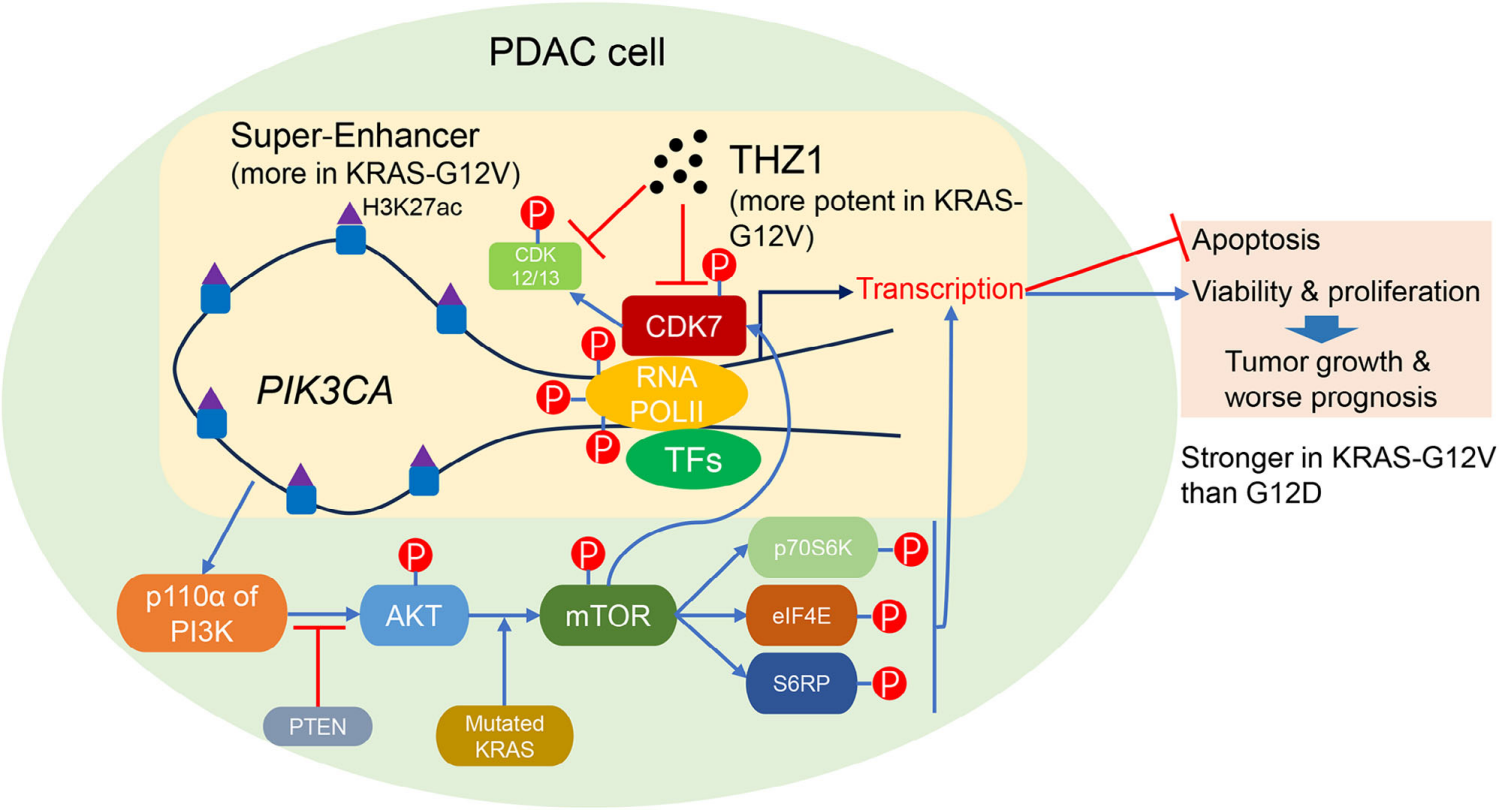
**Schematic diagram**. This hypothesis‐generating study suggests that THZ1 might have a stronger inhibitory effect on some pancreatic ductal adenocarcinomas (PDACs) bearing KRAS‐G12V mutation than some others with G12D mutation. Using certain cell lines, KRAS‐G12V cells had more H3K27ac‐binding super‐enhancers, and THZ1 significantly weakened the binding of H3K27ac to *PIK3CA* in KRAS‐G12V PDAC. Suppressive effect of THZ1 on phosphorylation of RNAPOLII, on super‐enhancer activity and H3K27ac binding to *PIK3CA*, and on PI3K/AKT/mTOR pathway and its downstream markers were all stronger in KRAS‐G12V than KRAS‐G12D PDAC. This study highlights the importance of differences in super‐enhancer activity and transcription regulation between discrepant KRAS mutations, regarding the highly selective suppressive efficacy of THZ1 on PDAC. Our findings that THZ1 might more potently inhibit certain KRAS‐G12V PDACs may offer novel key clues for precise management of PDAC and important evidence for future targeted trial design. Notably, this study suggests only an association rather than a causative relationship. Further validations through investigations involving a broader spectrum of PDAC cell types with KRAS‐G12V or KRAS‐G12D mutations are warranted.

In this study, we have unveiled a novel insight: The suppressive effectiveness of THZ1 on PDAC could potentially hinge on specific *KRAS* mutation subtypes, which might be associated with the influences on SE activity and the PI3K/AKT/mTOR pathway (Figure 6). PDAC is marked for distinctively high mortality rate and limited treatment avenues, and the therapy largely relies on drug‐based approaches.^50^ Conventional chemotherapy has seemingly plateaued, and targeted interventions such as bevacizumab, cetuximab and sunitinib have encountered setbacks, in part due to the intricate genetic landscape that complicates the identification of specific driver genes.^32^ Despite the pronounced heterogeneity within PDAC, a significant number of cases exhibit a profound reliance on transcription processes,^51^ suggesting that transcription suppression could prove an effective strategy against PDAC.

CDK7, an integral player within the general transcription factor TFIIH, exerts dual roles in cancer cell biology. It can not only promote transcription (initiation and pause) through RNAPOLII CTD phosphorylation but also influence the cell cycle, thereby expediting tumour initiation and progression.^16^, ^52^ In breast cancer, a high expression of *CDK7* mRNA was linked to a poorer prognosis, while overall CDK7 protein expression showed no significant prognostic link.^53^ In estrogen receptor (ER)‐positive^54^ and triple‐negative^53^ breast cancers, a high expression of CDK7 protein correlated with worse survival. In gastric carcinoma, high CDK7 protein expression significantly predicted an unfavourable prognosis.^55^, ^56^ Our findings indicated that low *CDK7* mRNA expression was associated with improved OS and DFS in PDAC. Notably, at the protein level, low CDK7 expression was associated with enhanced survival specifically in *KRAS‐G12V* PDAC but not in *KRAS‐G12D* cases, following multivariable adjustment. These observations suggest that CDK7 inhibition could hold promise as an effective *KRAS* mutation‐dependent therapeutic avenue against PDAC.

Our further investigation underscored abundant CDK7 expression within PDAC cells. *CDK7* knockout using CRISPR/CAS9 induced significant declines in PDAC cell viability and proliferation. The apoptotic marker cleaved‐PARP exhibited substantial elevation, while the antiapoptotic protein MCL1, a BCL‐2 family member and a mitochondrial apoptosis inhibitor often overexpressed in cancers, showed a notable reduction. The short half‐life of MCL1 makes it susceptible to transcription inhibition.^57^, ^58^ Our findings confirmed that targeting CDK7 in PDAC could effectively restrain proliferation and foster apoptosis, a pattern mirrored in T‐ALL, ovarian cancer, triple‐negative breast cancer and small‐cell lung cancer.^22^, ^28^, ^29^, ^59^ These results consolidate the idea that the development of PDAC is heavily reliant on the transcription regulation by CDK7.

In 2014, Kwiatkowski et al.^22^ demonstrated that the active acrylamide group of THZ1 could covalently bind to the cysteine residue Cys312 within the ATP‐binding domain of CDK7. This interaction potently inhibited cancer cell proliferation majorly by weakening the RNAPOLII‐occupied promoters and dampening transcription.^52^ THZ1 promotes apoptosis in oesophageal squamous cell carcinoma and small‐cell lung cancer.^7^, ^29^ Our investigation highlighted that PDAC cells were highly sensitive to THZ1, leading to dose‐dependent and time‐dependent increases in apoptosis across PDAC cell lines (CAPAN2, PANC03.27, SW1990 and PANC02.03). Remarkably, this apoptosis‐inducing effect proved notably more pronounced in *KRAS‐G12V* PDACs compared with *KRAS‐G12D* cases. These findings suggested that the propensity of THZ1 to promote apoptosis within PDAC cells was contingent upon the specific *KRAS* mutation status. This was further supported by results from both in vivo CDX and mini‐CDX model experiments. The highly selective PDAC‐inhibitory role of THZ1 may be intimately linked to the differential transcription activation mechanisms present within distinct *KRAS* mutation subtypes. Collectively, THZ1 might be a promising candidate for the selective treatment of *KRAS‐G12V* PDAC, potentially offering heightened potency.

As a component of cell cycle‐regulating kinase, CDK7 can phosphorylate and activate all the other CDKs, including cell cycle‐associated CDKs.^60^ CDK7 can promote mitosis, and is necessary for G2/M and G1/S phase activation via promoting the formation of the CDK1/cyclin‐B and CDK2/cyclin‐E complexes, respectively.^61^, ^62^ THZ1 blocked cells in the G2/M phase in *MYCN*‐amplified neuroblastoma^63^ and oesophageal squamous‐cell carcinoma.^7^ In ovarian cancer, the proportion of the S phase decreased after THZ1 treatment.^59^ However, in a study of triple‐negative breast cancer,^28^ THZ1 did not appear cell cycle‐blocking or affect the mitotic phase. We did not find that THZ1 significantly blocked the cell cycle in PDACs of both *KRAS* mutation subtypes. In cells irrespective of *KRAS* mutations, cyclin H level did not significantly change after adding THZ1. We speculate that the inhibition of THZ1 on PDAC may not be caused by CDK7 regulating other cell cycle‐related CDKs.

Continuing our exploration, we verified that THZ1 profoundly repressed global transcription in PDAC, which was closely associated with its effect on RNAPOLII. Furthermore, THZ1 significantly curbed the expression and phosphorylation (particularly at ser5) of RNAPOLII in PDAC, with a notably more pronounced effect in *KRAS‐G12V* cells. CDK7 can directly or indirectly (by phosphorylating other CDKs) influence RNAPOLII CTD phosphorylation at multiple sites (e.g. ser2, ser5 and ser7).^16^, ^17^, ^52^, ^61^, ^64^ THZ1 covalently binds to CDK7, which can lead to a dose‐dependent decrease in RNAPOLII CTD phosphorylation. Dephosphorylated RNAPOLII fails to form a transcription complex with histones, rendering it unable to bind to enhancer regions on DNA, which in turn inhibits the transcription of cancer cells. This could be corroborated by a noticeable reduction in DNA‐histone binding observed through ChIP assays. Our findings in PDAC are consistent with those in small‐cell lung cancer and triple‐negative breast cancer, where THZ1 can result in an extensive transcription reduction, possibly linked to SE activity.^7^, ^28^, ^29^, ^59^

SEs represent large clusters of multiple enhancers characterized by the enrichment of H3K27ac.^8^ H3K27ac is a pivotal histone regulating DNA replication, recombination, repair, chromatin stability, cell cycle and epigenetic enhancement.^65^ After combining with TFIIH and other transcription factors, the abnormally activated RNAPOLII in cancer cells can bind with specific histones on rRNA (such as H3K27ac) to form transcription complexes. The complexes can then bind with promoter and enhancer regions on DNA, resulting in the abnormal transcription activation of oncogenes. SEs play a crucial role in sustaining robust transcriptional activation of key oncogenes vital to PDAC development and progression.^66^ *RNAPOLII* expression is intrinsically linked to SEs in PDAC.^67^ Oncogenes associated with or driven by SEs were highly susceptible to CDK7 inhibition in PDAC, underscoring the potential of transcription‐disrupting strategies by targeting SEs to suppress PDAC.^8^, ^68^ We found that *KRAS‐G12V* PDAC cells exhibited a higher count of H3K27ac‐binding SEs. Remarkably, THZ1 significantly suppressed SE activity only in *KRAS‐G12V* cells, but not in *KRAS‐G12D* cells. Conversely, THZ1 exhibited more pronounced suppression of TEs in *KRAS‐G12D* cells.

Furthermore, our investigations unveiled that THZ1 significantly inhibited histone H3K27ac binding to *PIK3CA* in *KRAS‐G12V* cells via ChIP‐seq analysis, which sheds light on protein binding to specific gene sites. This supports that THZ1 regulates CDK7‐mediated transcription in PDAC, and indicates that PIK3CA may be a downstream regulatory target of THZ1. Our study found an inverse association between *PIK3CA* and prognosis. *PIK3CA* encodes p110α, the catalytic subunit of PI3K,^69^ which can activate AKT and its downstream transcription factors including mTOR via phosphorylation.^70^ Enhanced PIK3CA signalling also triggers CDK7 phosphorylation.^71^ mTOR, a pivotal serine/threonine kinase, governs cell metabolism and growth. mTOR regulates the activation of eukaryotic translation initiation factor 4E binding proteins (eIF4E), ribosomal kinase p70S6K and S6RP, all closely involved in transcription, translation and cellular protein synthesis.^72^

Accordingly, we observed that THZ1 effectively suppressed the PI3K/AKT/mTOR pathway, which was significantly associated with *CDK7* expression in PDAC, and the downstream molecules. Post THZ1 treatment, phosphorylation levels of AKT and mTOR were markedly reduced, along with the phosphorylation levels of downstream mTOR targets, including p70S6K, S6RP and eIF4E. This inhibitory effect was markedly stronger in *KRAS‐G12V* cells. In mutant *KRAS*‐driven PDAC cells, knocking out the transcriptional regulator CDK7 was as potent as knocking out *KRAS*.^33^ More than 80% of PDAC cases exhibit *KRAS* mutations, which may trigger various pathways, including the PI3K/AKT/mTOR signalling,^73^, ^74^ regulating cell cycle, proliferation, migration, differentiation, metabolism, transcription, protein synthesis, gene stability and radiation resistance.^75^ The PI3K signalling cascade plays a pivotal role in the carcinogenesis instigated by *KRAS* mutations in PDAC.^73^ Approximately half of PDAC patients exhibit abnormal PI3K signalling activation and AKT phosphorylation, often associated with poorer differentiation and worse prognosis.^76^, ^77^ Together, THZ1 can inhibit the PI3K/AKT/mTOR pathway activity by inhibiting SE‐regulated *PIK3CA*, which might be dependent on *KRAS* mutation.

In summary, this hypothesis‐generating study suggests that THZ1 has promising therapeutic potential against PDAC, which might be dependent on *KRAS* mutation status. THZ1 might selectively inhibit certain PDACs with *KRAS‐G12V* mutation more potently compared with some other PDACs with *KRAS‐G12D* mutation, which might be associated with its effects on SE activity and the PI3K/AKT/mTOR pathway. Notably, this study suggests only an association rather than a causative relationship. Further validations through investigations involving a broader spectrum of PDAC cell types with *KRAS‐G12V* or *KRAS‐G12D* mutations are warranted. These findings might pave the way for novel insights into the precise management of PDAC and serve as valuable evidence for the design of targeted anti‐PDAC trials in the future.

## AUTHOR CONTRIBUTIONS

*Conception and design*: Huang L, Dai G, Gu M and Shi Y.

*Development of methodology*: Huang L, Yang H, Chen K, Gu M and Shi Y.

Acquisition of data (acquired and managed patients, provided facilities, etc.): Huang L, Yang H, Chen K, Yuan J, Li J, Dai G, Gu M and Shi Y.

Analysis and interpretation of data (e.g. statistical analysis, biostatistics, computational analysis): Huang L, Yang H, Chen K, Dai G, Gu M and Shi Y.

*Writing, review and/or revision of the manuscript*: Huang L, Yang H, Chen K, Yuan J, Li J, Dai G, Gu M and Shi Y.

*Administrative, technical or material support (i.e. reporting or organizing data, constructing databases)*: Huang L, Dai G, Gu M and Shi Y.

*Study supervision*: Huang L, Dai G, Gu M and Shi Y.

All authors approved the manuscript for submission and publication.

## FUNDING INFORMATION

This study was funded by grants from the Start‐up Fund for the Introduction of High‐Level Talents by Ruijin Hospital, Shanghai Jiao Tong University School of Medicine. The funder had no role in study design; in the collection, analysis or interpretation of data; in the writing of the report; or in the decision to submit the paper for publication.

## ETHICAL APPROVAL STATEMENT

All samples were anonymously coded according to the local ethical guidelines as stipulated by the Declaration of Helsinki. Written informed consent was obtained from all the patients, and this study was approved by the Ethics Committee of Chinese PLA General Hospital.

## Supporting information

Supplementary Materials

## Data Availability

Restrictions apply to the availability of the Chinese data for this study, which were used under license, and so are not publicly available. The Cancer Genome Atlas dataset could be obtained from https://www.cancer.gov/about‐nci/organization/ccg/research/structural‐genomics/tcga with relevant permissions.
