# Erratum for the research article “The suppressive efficacy of THZ1 depends on KRAS mutation subtype and is associated with super‐enhancer activity and the PI3K/AKT/mTOR signalling in pancreatic ductal adenocarcinoma: A hypothesis‐generating study” by Huang et al

**DOI:** 10.1002/ctm2.70158

**Published:** 2024-12-27

**Authors:** Lei Huang, Hui Yang, Kaidi Chen, Jing Yuan, Jie Li, Guanghai Dai, Mancang Gu, Yan Shi

**Affiliations:** ^1^ Department of Oncology Ruijin Hospital, Shanghai Jiao Tong University School of Medicine Shanghai China; ^2^ School of Pharmaceutical Science Zhejiang Chinese Medical University Hangzhou China; ^3^ Department of Pathology Chinese PLA General Hospital Beijing China; ^4^ Department of Medical Oncology Chinese PLA General Hospital Beijing China; ^5^ Academy of Chinese Medical Sciences Zhejiang Chinese Medical University Hangzhou China; ^6^ Department of General Surgery Shanghai Seventh People's Hospital Shanghai University of Traditional Chinese Medicine Shanghai China

1

Some inadvertent mistake in our paper entitled “The suppressive efficacy of THZ1 depends on KRAS mutation subtype and is associated with super‐enhancer activity and the PI3K/AKT/mTOR signalling in pancreatic ductal adenocarcinoma: A hypothesis‐generating study” ^[^ ^1^ ^]^ and published in *Clinical and translational medicine* came to our attention recently:

(1) There was an unintentional mistake in the placement and assembly of representative individual bioluminescence images in the original Figure 2A, which does not impact the statistical analysis, the described findings, or the conclusions. We would like to correct the representative individual bioluminescence image in the red block of the original Figure 2A as (Figure 1):

**FIGURE 1:**
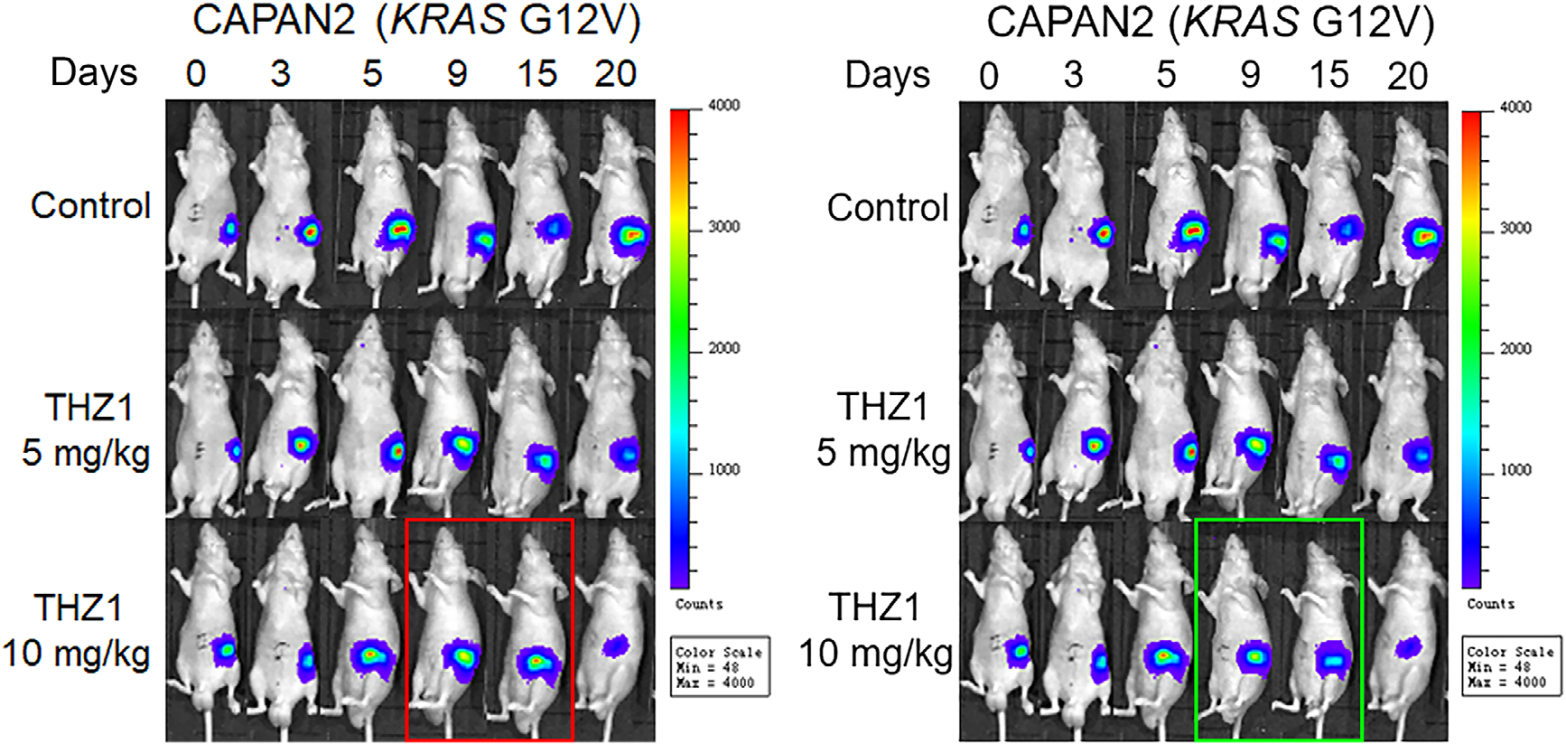
Correction for the original Figure 2A. The original figure is shown in the left panel with a red block, and the corrected figure is shown in the right panel with a green block.

(2) There was an unintentional mistake in loading control band placement in Western blot, which does not influence the described findings or the conclusions. We would like to correct the loading control GAPDH in the red block of the original Figure 1I as (Figure 2).

And correct the loading control GAPDH in the red block of the original Figure 5C and E as (Figure 3):

The corrections do not have any impact on the described findings, the conclusions, or any other part of the manuscript. We sincerely apologize for any inconvenience caused.

**FIGURE 2:**
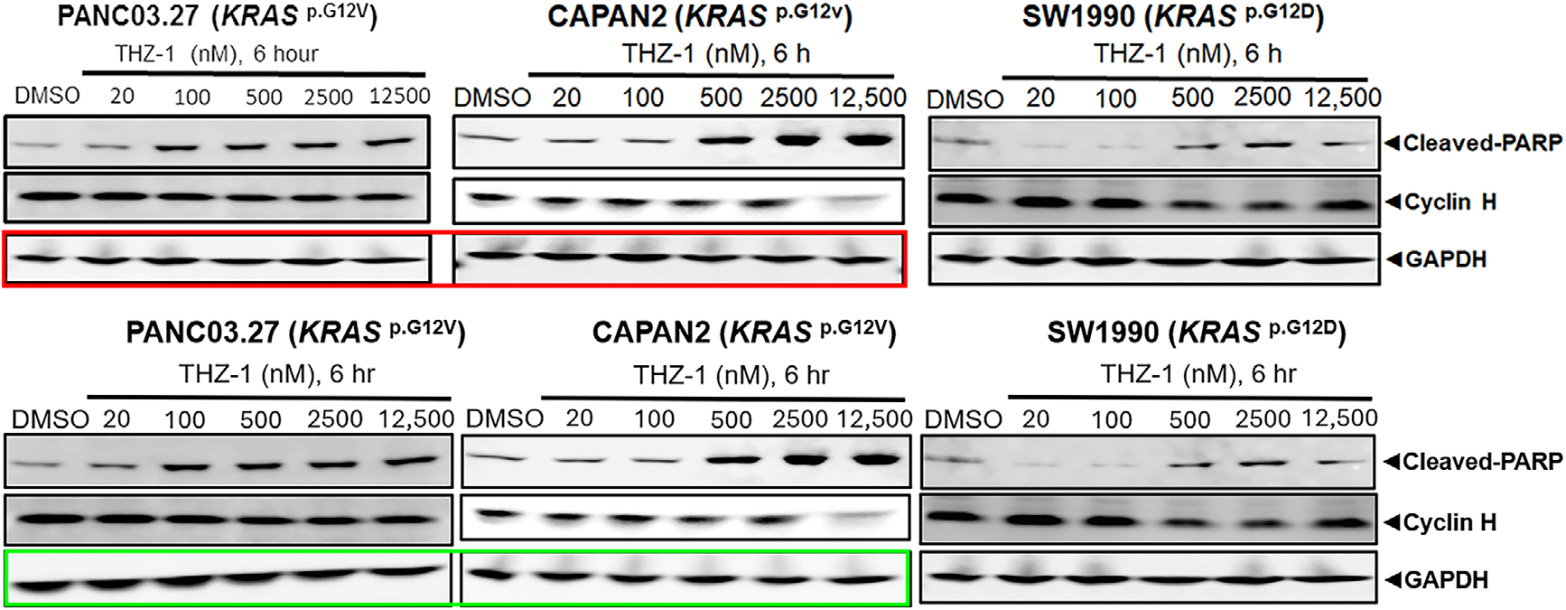
Correction for the original Figure 1I. The original figure is shown in the upper panel with a red block, and the corrected figure is shown in the lower panel with a green block.

**FIGURE 3:**
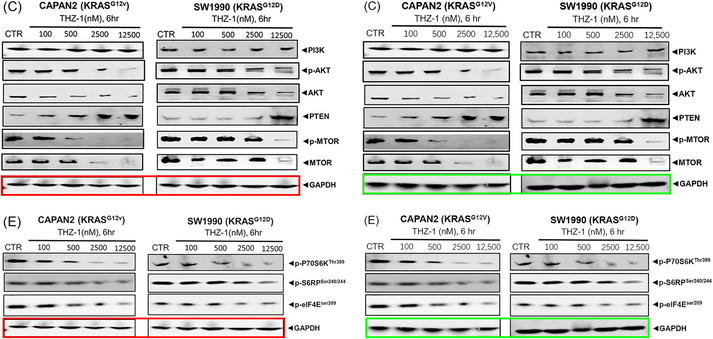
Correction for the original Figure 5C and E. The original figure is shown in the left panel with a red block, and the corrected figure is shown in the right panel with a green block.
