# Supplementary material for: The suppressive efficacy of THZ1 depends on KRAS mutation subtype and is associated with super‐enhancer activity and the PI3K/AKT/mTOR signalling in pancreatic ductal adenocarcinoma: A hypothesis‐generating study
Source: Clin Transl Med. 2023 Nov 30;13(12):e1500. doi: 10.1002/ctm2.1500 (PMC10689978; doi:10.1002/ctm2.1500)
Supplement: Supplementary file 1 — Supplementary Materials [file CTM2-13-e1500-s001.docx]

**Supplementary materials**

**Supplementary Methods**

***Reagents***

***Chromatin immunoprecipitation (ChIP)***

**Table S1.** Primers used for PCR of ChIP products

**Table S2.** Concentrations of DNAs obtained by ChIP-PCR

**Table S3**. Patient and tumor characteristics and biomarkers in our resected pancreatic cancer cohort, overall and stratified by *KRAS*

**Figure S1.** Effects of CDK7 knockout using CRISPR/CAS9 on the phosphorylation of RNAPOLII CTD at ser2, ser5, and ser7 in pancreatic ductal adenocarcinoma cells with different *KRAS* mutations

**Figure S2.** Inhibitory effects of THZ1 and YKL-05-124 on pancreatic ductal adenocarcinoma cells with different KRAS mutations

**Figure S3.** Gene Ontology (GO) enrichment analyses.

**Figure S4.** The inhibitory effect of treatment with 100 nM THZ1 for different time (control, 3, 6, 12, and 24 hours) on the expression and phosphorylation of the CTD of the transcription-related protein RNAPOLII in PANC03.27 (*KRAS-G12V*), CAPAN2 (*KRAS-G12V*), and SW1990 (*KRAS-G12D*) PDAC cell lines

**Figure S5.** Effect of THZ1 on the binding of H3K27ac with *PIK3CA* in SW1990 cells with *KRAS-G12D* mutation using *PI3KCA*-specific and negative control primers, respectively

**Supplementary Methods**

***Reagents***

Anti-CDK7, anti-RNAPOLII, anti-GAPDH, A-tubulin (loading control), and puromycin were purchased from Santa Cruz Biotechnology, and anti-phosphorylated-CTD-RNAPOLII-ser2/5/7 from Millipore. Anti-cleaved-PARP (Asp214) was purchased from Cell Signaling Technology, and anti-cyclin H and anti-MCL1 from Abcam. H3K27ac antibody was purchased from Abcam, and Caspase 3/7 from Promega Corporation. Alexa680-conjugated anti-rabbit IgG was bought from Invitrogen, and IRDye800-conjugated anti-mouse IgG from Rockland. PI/RNASE stain and ANXN V FITC apoptosis kit were bought from Thermo Fisher Scientific, and crystal violet dye from Sigma Aldrich. Trypsin, phosphate-buffered saline (PBS) buffer, MAGnify Chromatin Immunoprecipitation System, Histone H3 (acetyl K27) antibody, H3K9me antibody, IgG antibody, Platinum SYBR Green qPCR Super Mix, custom primers, and 1.5%-2.0% agarose gel were bought from Life Technologies. The RNeasy Plus Mini Kit was obtained from Qiagen.

***Chromatin immunoprecipitation (ChIP)***

*Cell crosslinking*

Cells were trypsinized, washed with 10 mL PBS, and then prepared into a 500-μL suspension containing 1×10^7^ cells. 13.5 μL formaldehyde was added for fixation (final concentration, 1%), followed by mixing and incubation at room temperature for 10 minutes. 57 μL 1.25 M glycine at room temperature was added to terminate the reaction, followed by mixing and incubation for 5 minutes at room temperature. The mixture was centrifuged for 10 minutes (4 °C, 200 *g*), and then placed on ice. Approximately 30 μL supernatant was retained in the centrifuge tube, and the rest was removed. The retained supernatant was resuspended using 500 μL cold PBS and centrifuged again for 10 minutes (4 °C, 200 *g*). Subsequently, 10-20 μL supernatant was retained while the rest was removed.

*Cell lysis*

The cell lysis buffer was prewarmed to room temperature and supplemented with a protease inhibitor. To every 1×10^7^ cells, 50 μL of cell lysis buffer containing the protease inhibitor was added, followed by mixing. The mixture was centrifuged at 4 °C for 15 minutes and placed on ice for at least 5 minutes.

*Cleaving of chromatin complex DNA using ultrasound*

A 1.5-mL Lobind tube was prepared, and the ultrasonic cutting machine (Pico diagenode) was precooled to 4 °C. The number of cycles was set to 15, with 30-second on-time and 30-second off-time (instant separation and pause). After centrifugation at 4 °C for 5 minutes, the supernatant containing chromatin was obtained. After ultrasound treatment, the chromatin was added with Proteinase K, followed by trypsinization for 20 minutes at 55 °C and then electrophoresis in a 2% agarose gel to determine whether the ultrasound fragmented the DNA into segments of 150-900 bp. Accordingly, the ultrasound cycle was then adjusted.

*Magnetic bead preparation and chromatin dilution*

Magnetic beads were placed on ice, resuspended, and added with 100 μL cold diffusion buffer and 10 μL fully resuspended Dynabeads protein A/G, followed by mixing for 5 minutes. The tube was then placed on a magnetic frame with the liquid removed. Subsequently, 100 μL dilution buffer (200× protease inhibitor plus stock dilution buffer) was added. The chromatin was diluted (at a ratio of 1:3 to 1:4), and 10 μL input was retained until the crosslink was removed.

*Chromatin immuno-co-precipitation*

Antibody was added for binding followed by overnight incubation at 4 °C. The target antibody used was H3K27ac, with IgG serving as the negative control. The reagents included H3K9me3 antibody and SAT primer, which were employed as control to verify experiment accuracy.

*Elution and de-crosslinking*

IP Buffer 1 (100 μL) was added to each sample followed by rotation at 4 °C for 5 min. This elution step was repeated three times. Next, 100 μL IP Buffer 2 was added to each sample, followed by rotation at 4 °C for 5 minutes. This elution step was repeated twice.

53 μL stock reverse crosslinking buffer and 1 μL Proteinase K were prepared into a 54-μL mixture. Each sample was added with a 54-μL mixture and placed at 55 °C for 15 minutes. The liquid was then transferred into a new tube and placed at 65 °C for 15 minutes and then on ice for 5 minutes. Subsequently, the magnetic bead was discarded.

*DNA purification*

A 70-μL mixture of DNA purification magnetic beads (50 μL DNA purification buffer plus 20 μL DNA magnetic beads) was prepared. For each mixture, 70-μL DNA purification magnetic beads were added, followed by incubation at room temperature for 5 minutes. The tube was placed on a magnetic rack, and the liquid was removed. Then, 150 μL DNA rinse buffer was added, followed by five rounds of washing and liquid removal.

*DNA dissolution*

DNA dissolution buffer (50-100 μL) was added to each mixture, followed by incubation at 55 °C for 20 minutes. The dissolved DNA was transferred into a new disinfected tube, which could be stored at -20 °C until further use.

The ChIP products were then subjected to a polymerase chain reaction (PCR) assay (**Tables S1-S2**).

**Table S1.** Primers used for PCR of ChIP products

| Sequence (5'->3') | Length | bp | Tm | GC% | nmol | ddH_2_O (100 uM) |
| --- | --- | --- | --- | --- | --- | --- |
| *RUNX1* |  |  |  |  |  |  |
| Forward primer | CCGAGAACCTCGAAGACATC | 21 | 57.8 | 55 | 28.9 | 289 |
| Reverse primer | GATGGTTGGATCTGCCTTGT | 20 | 57.87 | 50 | 23.9 | 239 |
| *SAT2* | | | | | | |
| Forward primer | GCTTCCTGCAAGAGTCGAAT | 20 | 58.27 | 50 | 23.9 | 239 |
| Reverse primer | ATTGGCTTCTCAAGATACCTG | 21 | 55.36 | 42.86 | 22.6 | 226 |
| *PIK3CA* | | | | | | |
| Forward primer | CTGTGTCTTCTGCACCAGGA | 20 | 59.61 | 55 | 26.4 | 264 |
| Reverse primer | AGAAGGACACATGGCTGGAC | 21 | 59.67 | 55 | 27.5 | 275 |
| Negative region of *PIK3CA* | | | | | | |
| Forward primer | GATCTAGATATGCCCAGTTC | 20 | 52.52 | 45 | 43.23 | 432.3 |
| Reverse primer | CAGCCTTCCCAGGTACACTTG | 21 | 60.61 | 57.1 | 34.51 | 345.1 |
| *GAPDH* | | | | | | |
| Forward primer | ATCTCGCTCCTGGAAGATG | 19 | 56.3 | 52.63 | 22.6 | 226 |
| Reverse primer | TCGGAGTGAACGGATTCG | 18 | 56.81 | 55.56 | 19.4 | 194 |

**Table S2.** Concentrations of DNAs obtained by ChIP-PCR

| Sample name | Target | Original sample conc. | Units | Sample Volume (µL) |
| --- | --- | --- | --- | --- |
| CAPAN2_CTR | H3K27ac | 8.5 | ng/µL | 28 |
| CAPAN2_T500nM6H | H3K27ac | 5.34 | ng/µL | 28 |
| CAPAN2_CTR_H3K9me | H3K9me | 7.48 | ng/µL | 28 |
| CAPAN2_CTR_IgG | IgG | 1.27 | ng/µL | 28 |
| CAPAN2_CTR_INPUT | / | 24.6 | ng/µL | 28 |
| CAPAN2_T500nM6H_INPUT | / | 17.1 | ng/µL | 28 |

**Table S3**. Baseline patient and tumor characteristics, biomarkers, and survival in the resected pancreatic ductal adenocarcinoma cohort of the China PLA General Hospital, overall and stratified by *KRAS* mutants

| **Variables** | **Overall** | ***KRAS G12D*** | ***KRAS G12V*** | ***P*** |
| --- | --- | --- | --- | --- |
| ***Patient and tumor characteristics*** |  |  |  |  |
| n | 241 | 100 | 80 |  |
| Age (years) | 66±11; 67 (59-74) | 65±11; 66 (58-74) | 64±12; 66 (57-73) | 0.587 |
| Sex, male | 135 (56) | 57 (57) | 43 (54) | 0.663 |
| Tumor location |  |  |  | 0.657 |
| Pancreas head | 169 (70) | 77 (77) | 57 (71) |  |
| Pancreas body | 24 (10) | 6 (6) | 8 (10) |  |
| Pancreas tail | 32 (13) | 8 (8) | 10 (13) |  |
| Pancreas uncinate | 11 (4) | 7 (7) | 4 (5) |  |
| Overlapping lesions or NOS | 5 (2) | 2 (2) | 1 (1) |  |
| Tumor size (cm) | 3.5±1.3; 3.3 (2.5-4.0) | 3.4±1.0; 3.1 (2.6-4.0) | 3.5±1.4; 3.1 (2.5-4.0) | 0.747 |
| pT stage |  |  |  | 0.146 |
| T1 | 21 (9) | 4 (4) | 10 (13) |  |
| T2 | 153 (63) | 69 (69) | 47 (59) |  |
| T3 | 59 (24) | 24 (24) | 19 (24) |  |
| T4 | 8 (3) | 3 (3) | 4 (5) |  |
| pN stage, N1-2 | 167 (69) | 75 (75) | 53 (66) | 0.198 |
| Examined lymph node number | 18±11; 16 (10-24) | 18±11; 16 (11-23) | 19±11; 17 (10-24) | 0.474 |
| Positive lymph node number | 3±3; 2 (0-4) | 4±4; 2 (1-5) | 3±3; 2 (0-4) | 0.094 |
| Differentiation grade |  |  |  | 0.065 |
| Well-differentiated | 20 (8) | 4 (4) | 7 (9) |  |
| Moderately-differentiated | 123 (51) | 46 (46) | 47 (59) |  |
| Poorly-differentiated/undifferentiated | 91 (38) | 47 (47) | 23 (29) |  |
| Unknown | 7 (3) | 3 (3) | 3 (4) |  |
| Vascular invasion |  |  |  | 0.723 |
| No | 29 (12) | 10 (10) | 12 (15) |  |
| Major vascular invasion | 8 (3) | 3 (3) | 2 (3) |  |
| Non-major vascular invasion | 191 (79) | 82 (82) | 61 (76) |  |
| Unknown | 13 (5) | 5 (5) | 5 (6) |  |
| Lymphatic invasion |  |  |  | **0.036** |
| No | 110 (46) | 34 (34) | 42 (53) |  |
| Yes | 116 (48) | 60 (60) | 33 (41) |  |
| Unknown | 15 (6) | 6 (6) | 5 (6) |  |
| Perineural invasion |  |  |  | 0.589 |
| No | 26 (11) | 9 (9) | 11 (14) |  |
| Yes | 203 (84) | 85 (85) | 65 (81) |  |
| Unknown | 12 (5) | 6 (6) | 4 (5) |  |
| Resection type |  |  |  | 0.681 |
| Whipple procedure (pancreaticoduodenectomy) | 179 (74) | 81 (81) | 60 (75) |  |
| Distal pancreatectomy | 60 (25) | 18 (18) | 19 (24) |  |
| Total pancreatectomy | 2 (1) | 1 (1) | 1 (1) |  |
| Resection margin |  |  |  | 0.367 |
| R0 | 121 (50) | 53 (53) | 39 (49) |  |
| R1 | 115 (48) | 46 (46) | 39 (49) |  |
| R2 | 3 (1) | 0 (0) | 2 (3) |  |
| RX | 2 (1) | 1 (1) | 0 (0) |  |
| Neoadjuvant therapy, yes | 14 (6) | 2 (2) | 7 (9) | 0.080 |
| Adjuvant therapy |  |  |  | 0.747 |
| No | 64 (27) | 30 (30) | 20 (25) |  |
| Chemotherapy only | 63 (26) | 24 (24) | 24 (30) |  |
| Radiotherapy only | 15 (6) | 7 (7) | 3 (4) |  |
| Chemoradiotherapy | 78 (32) | 30 (30) | 26 (33) |  |
| Unknown | 21 (9) | 9 (9) | 7 (9) |  |
| ***Biomarkers*** |  |  |  |  |
| *KRAS* SNV |  |  |  | - |
| *G12D* | 100 (41) | 100 (100) | 0 (0) |  |
| *G12V* | 80 (33) | 0 (0) | 80 (100) |  |
| Other | 61 (25) | 0 (0) | 0 (0) |  |
| CDK7 expression score |  |  |  |  |
| As continuous | 8±3; 9 (6-9) | 8±3; 9 (6-9) | 8±3; 9 (6-9) | 0.506 |
| High expression | 153 (63) | 67 (67) | 48 (60) | 0.331 |
| ***Survival*** |  |  |  |  |
| Overall survival (months) | 20 (10-38) | 14 (9-26) | 24 (16-46) | **<0.001** |
| Disease-free survival (months) | 14 (7-32) | 10 (4-24) | 17 (8-33) | **0.003** |

Continuous data are shown as mean ± standard deviation; median (interquartile rage), and categorical variables as count (percentage [%]). Survival data are shown as median (interquartile rage).

NOS, not otherwise specified; SNV, single nucleotide variant.


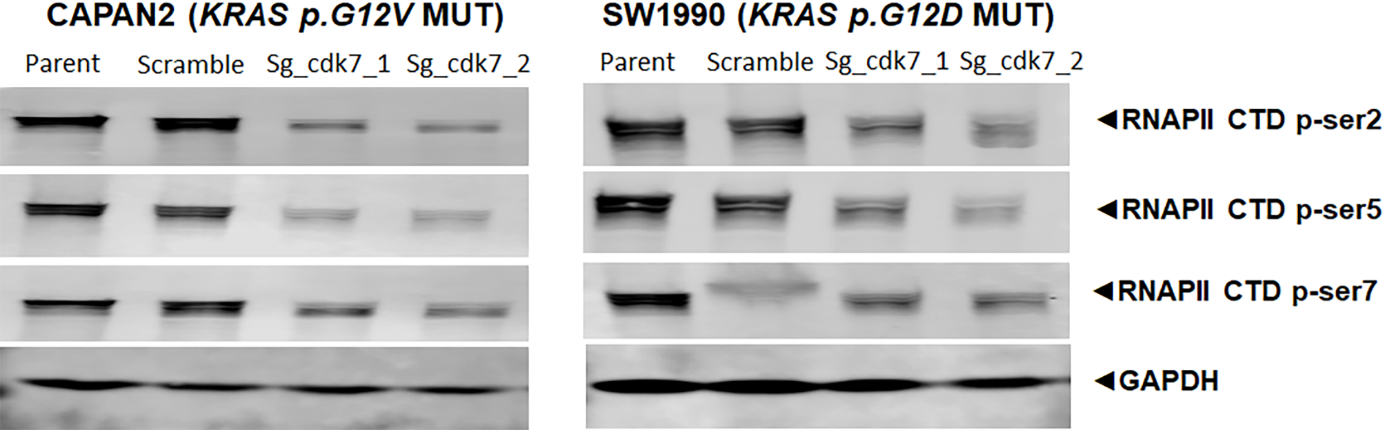


**Figure S1.** Effects of CDK7 knockout using CRISPR/CAS9 on the phosphorylation of RNAPOLII CTD at ser2, ser5, and ser7 in pancreatic ductal adenocarcinoma cells with different *KRAS* mutations.


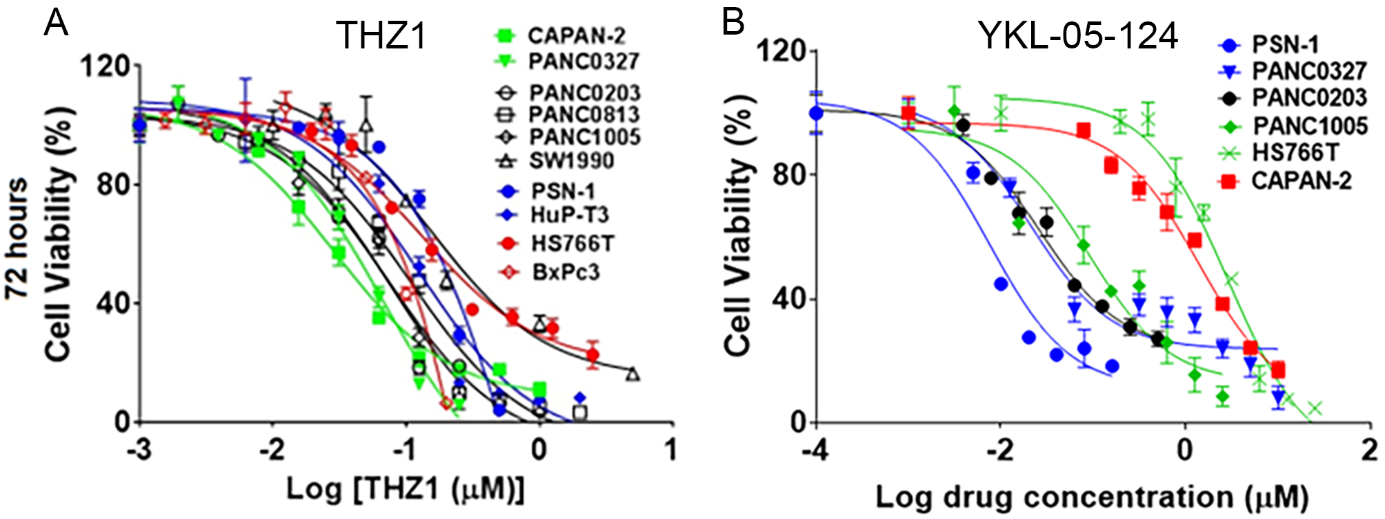


**Figure S2.** Inhibitory effects of THZ1 (**A**) and YKL-5-124 (**B**) on pancreatic ductal adenocarcinoma cells with different *KRAS* mutations. *KRAS-G12V*: CAPAN2 and PANC03.27 (green lines in **A**); *KRAS-G12D*: SW1990, PANC02.03, and PANC10.05


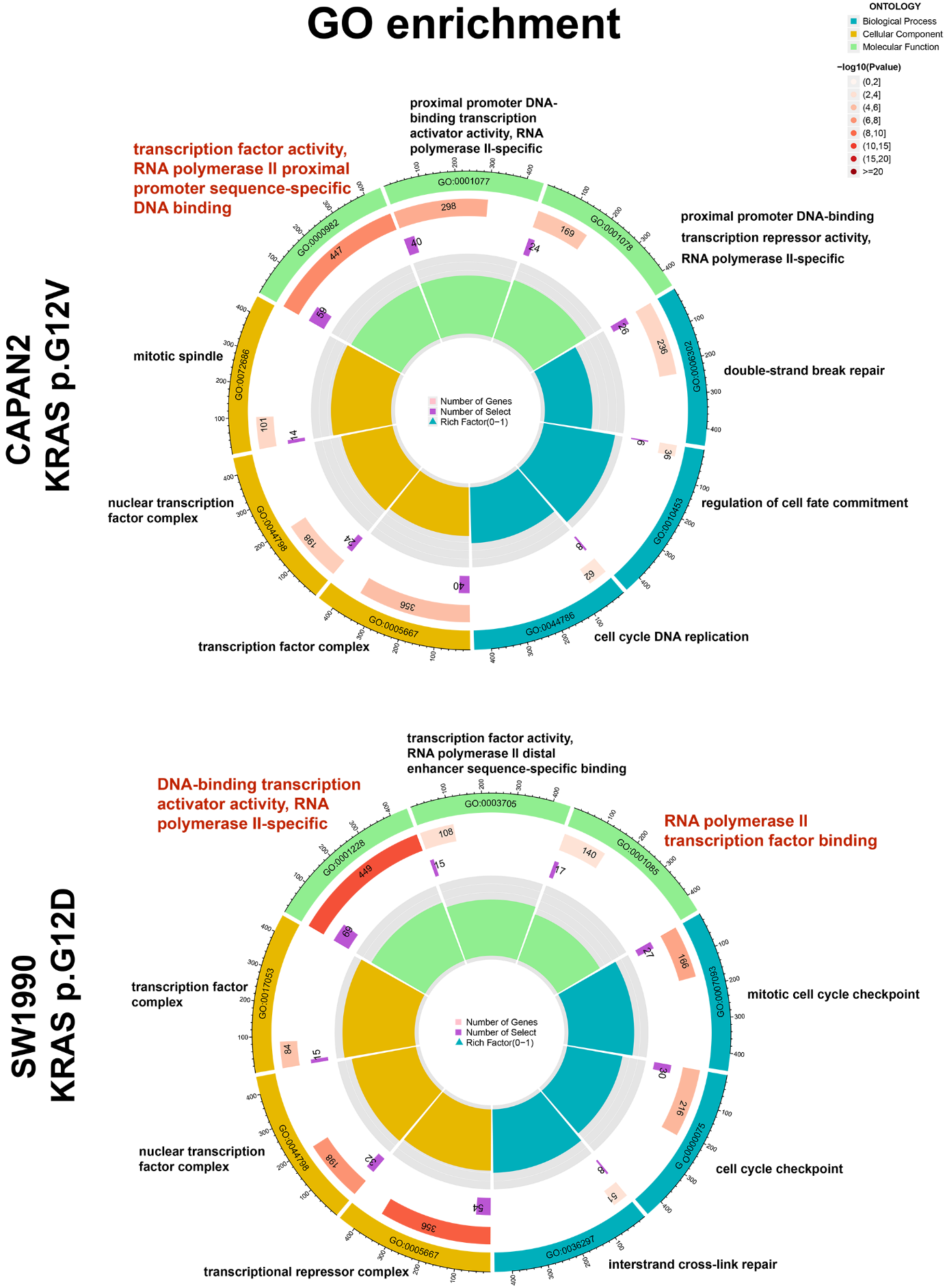


**Figure S3.** Gene Ontology (GO) enrichment analyses.





**Figure S4.** The inhibitory effect of treatment with 100 nM THZ1 for different time (control, 3, 6, 12, and 24 hours) on the expression and phosphorylation of the CTD of the transcription-related protein RNAPOLII in PANC03.27 (*KRAS-G12V*; **Left**), CAPAN2 (*KRAS-G12V*; **Middle**), and SW1990 (*KRAS-G12D*; **Right**) PDAC cell lines.


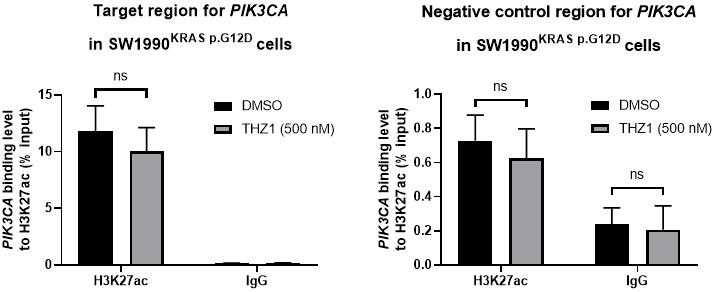


**Figure S5.** Effect of THZ1 on the binding of H3K27ac with *PIK3CA* in SW1990 cells with *KRAS-G12D* mutation using *PI3KCA*-specific and negative control primers, respectively. ns, not significant.
